# Mechanism of action of the third generation benzopyrans and evaluation of their broad anti-cancer activity *in vitro* and *in vivo*

**DOI:** 10.1038/s41598-018-22882-w

**Published:** 2018-03-23

**Authors:** Alexander J. Stevenson, Eleanor I. Ager, Martina A. Proctor, Dubravka Škalamera, Andrew Heaton, David Brown, Brian G. Gabrielli

**Affiliations:** 1 Mater Research Institute, The University of Queensland, Translational Research Institute, Brisbane, QLD Australia; 2 Novogen Ltd., Hornsby, New South Wales Australia; 3 Present Address: School of Medical Sciences, University of New South Wales Australia, Sydney, New South Wales Australia

## Abstract

Successive rounds of chemical modification in three generations of benzopyran molecules have shown to select for different mechanisms of actions and progressive increases in anti-cancer activity. In this study, we investigated the mechanism of action of the third-generation benzopyran compounds, TRX-E-002-1 and TRX-E-009-1. High-content screening of a panel of 240 cancer cell lines treated with TRX-E-009-1 demonstrated it has broad anti-cancer potential. Within this screen, melanoma cell lines showed a range of sensitivities and subsequently a second independent panel of 21 melanoma 3D spheroid lines were assessed for their responses to both TRX-E-002-1 and TRX-E-009-1 compounds. Time-lapse microscopy illustrated both of these compounds caused mitotic delays in treated cells, resulting in either mitotic slippage or apoptosis. This finding along with immunostaining, *in vitro* polymerization assays, and animal experiments in both athymic and immunocompetent mice, demonstrates that these third-generation benzopyran compounds are potent tubulin polymerization inhibitors *in vitro* and *in vivo*, and this is the molecular basis of their anti-cancer activity in melanoma. These findings indicate these BP compounds may offer a novel anti-microtubule strategy for cancer intervention and provides the basis for further investigation into biomarkers of clinical sensitivity.

## Introduction

The benzopyran pharmacophore has provided fertile ground for oncology drug candidates. Chemical optimization has resulted in three generations of benzopyran (BP) molecules. First-generation compounds showed promise in clinical trials but were limited by poor bioavailability^1–4^. Of the second-generation BPs, ME-344 remains under clinical investigation^5–7^. A third-generation of BPs, which includes Trilexium (TRX-E-009-1) and Cantrixil (TRX-E-002-1), have now been developed, with the latter currently under assessment in a Phase 1 trial. Preclinical studies indicate that with each generation, a progressive increase in anti-cancer activity has been achieved and that third-generation BP compounds are not only cytotoxic to differentiated cancer cells but undifferentiated/stem-like cancer cells^8–15^. Moreover, successive rounds of chemical modification have selected for different mechanisms of actions, reflecting the pleiotropic nature of the pharmacophore^8–15^. The increase in anti-cancer potency through the generations likely reflects changes in the predominant mode of action.

Mechanisms of action attributed to BP molecules include disruption of the sphingomyelin cycle associated with induction of caspase-mediated apoptosis (Phenoxodiol and Triphendiol), inhibition of topoisomerase II, degradation of the electron transport chain and reduction in ATP production leading to caspase-independent cell death (Triphendiol, ME-143, and ME-344) as well as activation of AMPK leading to mTOR mediated autophagy (ME-143), inhibition of tubulin polymerization (ME-344), and activation of cJUN (Cantrixil/TRX-E-002-1)^8–16^. The third generation BP, TRX-E-002-1 (formulated in the drug product Cantrixil), has cytotoxic activity to the undifferentiated stem-like cancer cells as well as the more differentiated (non-cancer stem cells) cancer cells^16^, possibly reflecting a unique mechanism of action or increased potency of an existing mechanism of action. Although the mechanism of cell death has been investigated for TRX-E-002-1, implicating JNK/JUN in caspase-mediated cell death, the molecular target/s have yet to be identified.

Here we investigated the mechanism of action of the third-generation BP compounds, TRX-E-009-1 and TRX-E-002-1. We report the broad anti-cancer activity of TRX-E-009-1 against a panel of over 200 cancer cell lines, and in 3D and animal models of melanoma. We demonstrate these compounds are potent tubulin polymerization inhibitors *in vitro* and *in vivo* and that this is the molecular basis of their anti-cancer activity in melanoma.

## Materials and Methods

### Reagents

TRX-E-009-1 and TRX-E-002-1 as well as the inactive racemic form of TRX-E-009 (TRX-E-009-2) were manufactured by GVK Biosciences and provided by Novogen Ltd. Nocodazole, Colchicine, DMSO, and resazurin were purchased from Sigma Aldrich. All other cell culture reagents were sourced from Life Technologies unless otherwise stated. All primary antibodies were from Cell Signaling Technologies and secondary antibodies from Life Technologies unless otherwise listed; rabbit anti-αTubulin (#ab18251; Abcam), rabbit anti-MEK1 (#ab32576, Abcam), mouse anti-αTubulin (#T6199, Sigma Aldrich), rabbit anti-GAPDH (#2275-PC-100, Trevigen), TRITC conjugated phalloidin (#P1951, Sigma Aldrich), DAPI (#BID0433, Apollo Scientific), goat anti-rabbit Alexa488 (#A11034), goat anti-mouse Alexa647 (#A21236), goat anti-rabbit Alexa555 (#A21428), rabbit anti-pMEK1 Thr286 (#9127), rabbit anti-Cleaved PARP (#9541), Rabbit anti-phospho-Histone 3 Ser10 (#9701).

### Cell Culture

All of the melanoma cell lines, except for D28 and A375, were sourced from Prof Nick Hayward’s lab at QIMR Berghofer as 2-dimensional cultures, then the 3-dimensional tumour sphere lines were derived from those. D28 cells were provided by Rick Pearson, Peter MacCallum Cancer Institute (Melbourne, Australia) and the A375 line was provided by Helen Rizzo, Westmead Institute for Cancer Research (Sydney, Australia). All 2-dimensional melanoma cell lines and primary human neonatal fibroblasts (NFF) were grown in RPMI1640 (Sigma Aldrich) supplemented 10% FBS (Bovogen), 2 mM L-Glutamine, 1 mM Sodium Pyruvate and 25 mM HEPES. All 3-dimensional melanoma tumour sphere cell lines were grown as described in^17^ without the addition of β-mercaptoethanol, in tissue culture flasks coated with 5 mg/ml Poly(2-hydroxyethyl methacrylate) (Sigma Aldrich). HeLa cells were grown in high glucose DMEM (Sigma Aldrich) supplemented with 10% FBS (Bovogen), 2 mM L-Glutamine, 1 mM Sodium Pyruvate and 25 mM HEPES. All cell lines were authenticated by STR profiling (Australian Genome Research Facility) and confirmed mycoplasma negative by the MycoAlert kit (Lonza).

### Eurofins Oncopanel Activity Data

The cytotoxic activity of TRX-E-009-1 was investigated against Eurofins’ OncoPanel240 (Eurofins, Missouri, USA). Cells were seeded into 384 well plates in standardized media and were allowed to attach overnight prior to treatment. TRX-E-009-1 was diluted in DMSO at a top concentration of 30 μM and then serially diluted in DMSO by 3.16-fold to complete a 10-point concentration curve. DMSO at 0.1% provided a control. Dilutions of TRX-E-009-1 were added to cell plates using Echo 550 acoustic energy based transfer and cells incubated for 72 hours. Cells were then fixed and stained to visualize nuclei, apoptotic and mitotic cells. Apoptotic cells were detected using an anti-cleaved caspase 3/7 antibody. Mitotic cells were detected using an anti-phospho-Histone 3 antibody, and DAPI staining was used to visualize nuclei.

Cellular response parameters were calculated using nonlinear regression to a sigmoidal single-site dose response model. IC_50_, defined as the test compound concentration at 50% of the maximum possible response, and cell count activity area, an estimate of the integrated area above the response curve, was calculated.

### Dose Response Experiments

Dose responses to TRX-E-009-1 and TRX-E-002-1 were performed using 3D tumour sphere cultures of 21 melanoma cell lines. Cells were dissociated and seeded^17^ at previously optimized densities into 384-well Ultra-Low Attachment plates (#3827, Corning). Cells were treated the following day with a 7 point 3-fold dilution series, 5.4 µM to 7.4 nM using a Sciclone ALH 3000 Liquid handling robot. Changes in cell viability were assayed at 72 hours using the Cell Titre Glo 3D assay (#G9683, Promega), luminescence reads were performed on a SynergyMx Plate Reader (BioTek Instruments). Luminescence values were normalized to the DMSO/vehicle control prior to comparisons.

### Time-Lapse Microscopy

Melanoma cells (A15, A2058, D04, SKMEL13, and SKMEL28) were seeded in 12-well plates (#3513, Corning) and incubated overnight prior to treatment with 300 nM TRX-E-009-1 or vehicle control. Once treated, cells were immediately set up for time-lapse microscopy using an Olympus CellR live cell microscope equipped with an incubation chamber at 37 °C and 5% CO_2_ and Cell Sens Software. Images were captured at 30 minute intervals and a minimum of 150 cells per condition were analysed for their time in mitosis.

### Tubulin Polymerization and Colchicine Competition Assays

The effect of the TRX-E-009-1 and TRX-E-002-1 on tubulin polymerization *in vitro* was assessed via a Tubulin Polymerization Assay Kit (#BK006P, Cytoskeleton) as per manufacturers’ instructions. A variation of the kit assay to measure the competitive binding of the compounds against Colchicine was performed as described in^11^. Both assays were read on a FLUOstar Optima (BMG Labtech), the tubulin polymerization assay having absorbance at 340 nm read every minute for 60 minutes and the Colchicine competition assay being an endpoint fluorescence read after 60 minutes at 355 nm excitation and 460 nm emission.

### Animal Husbandry

All animal studies were conducted at Jubilant Biosys (#96, Industrial Suburb, Yeshwanthpur, Bangalore-560022, India). The facility where the experiments were performed is AAALAC (Association of Assessment and Accreditation of Lab Animal Care International) accredited. Procedures involving the care and use of animals in this study were reviewed and approved by the Institutional Animal Care and Use Committee (IAEC/JAC/2012-2032 and IAEC/JAC/2013-44). During the study, the care and use of animals was in accordance with the principles outlined in the Guide for the Care and Use of Laboratory Animals 8^th^ Edition, 2010 (Indian National Research Council). Animals were given an autoclaved commercial diet (Nutrilab Rodent Feed, cylindrical shaped pellets) and autoclaved water *ad libitum*. Animals were assessed for clinical condition, body weight loss and tumour burden in accordance with ethical guidelines.

### Xenograft Studies

A375 cells were suspended in serum free medium at a concentration of 5 million cells/100 µl then mixed in a 1:1 ratio with Matrigel. Athymic mice were inoculated with the A375 cells/matrigel mix subcutaneously to induce tumours. Once tumour volume reached ~130 mm^3^, mice were assigned into control or treatment groups (n = 8 mice per group). TRX-E-009-1 in a solutol mixture (10 parts of 5% dimethylacetamide (DMA) in PBS + 90 parts of 10% Solutol in PBS) was administered via an intravenous (IV) injection daily at a dose of 60 mg/kg. Dabrafenib was dosed orally at 30 mg/kg in 0.5% hydroxypropylmethylcellulose (HPMC) + 0.2% Tween-80 in pH 8 (98:2). The doses of TRX-E-009-1 and Dabrafenib used in combination were the same as the monotherapy doses used for each agent. The solutol mixed was delivered by IV daily for use as a control and all treatments were 15 days.

To assess the *in vivo* activity of TRX-E-009-1, a second set of A375 xenografts were allowed to reach ~150 to 200 mm^3^ then treated with 80 mg/kg TRX-E-009-1 or vehicle control by IV daily for 5 days. Tumours were excised 4 hours after the final dose and formalin fixed prior to paraffin embedding.

B16-F10 cells (0.1 million viable cells, total volume with Matrigel of 100 μl) were implanted subcutaneously in the right dorsal flank of C57/BL6 mice. When the tumour volume reached ~75 mm^3^ animals were randomized into groups (n = 8 mice per group). TRX-E-009-1 in solutol mixture was administered via tail vein injection at 5 and 60 mg/kg body weight. Solutol mixture was administered via IV injection as vehicle control. All treatments were for administered daily for 15 days. Tumour growth continued to be monitored over the treatment period. A tumour volume of 2000 mm^3^ was considered the ethical limit for tumour burden and, if reached, mice were sacrificed without the need for any other signs of morbidity.

### Immunofluorescence of Xenograft Tumour Sections

Formalin-fixed paraffin-embedded xenograft sections were deparaffinised and rehydrated through xylene to water. Antigen retrieval was performed in 10 mM citrate buffer pH 6.0 in a commercial decloaking chamber (Biocare Medical) for 5 minutes at 125 °C. Non-specific binding was blocked with 0.1% saponin/3% bovine serum albumin (Sigma Aldrich) in TBS for 60 min. Primary antibodies were incubated overnight at 4 °C prior to detection with Alexa Fluor conjugated secondary antibodies. Sections were washed in TBS, mounted and cover-slipped using ProLong Gold Anti-fade with DAPI reagent (Life Technologies).

### Statistical Analysis

All statistical calculations were performed using Prism 5.0 or later (GraphPad Software Inc, USA). Comparisons of tumour size and body weight during and at the termination of the study were made between the treatment groups and respective vehicle control groups by two-way ANOVA, followed by Bonferroni’s correction for multiple comparisons. For survival analyses a Log rank test (Mantel-Cox) was performed. A p-value less than 0.05 was considered significant. For mitotic phase object counts an unpaired t-test with Welch’s correction was performed between TRX-E-009-1 and Vehicle for each phase.

### Data availability

Movies of the data presented in Fig. 3 can be found at 10.6084/m9.figshare.5789706.

## Results

### TRX-E-009-1 Shows Broad Spectrum Effectivity

**Figure 1:**
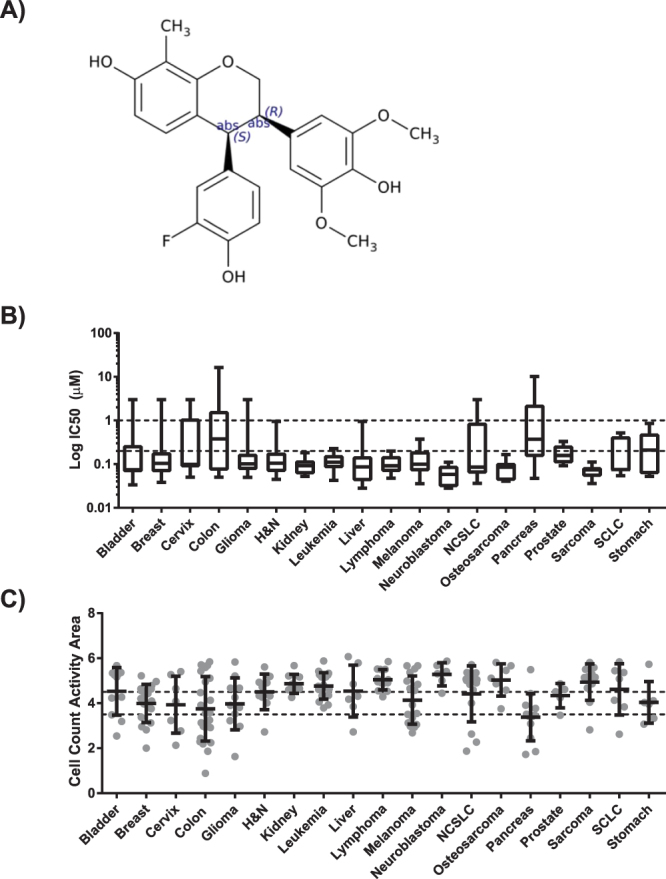
TRX-E-009-1 compound structure (**A**) and its effect on viability of cancer cell lines expressed as IC_50_ (**B**) or cell count activity area (**C**) (combined score for IC_50_ and fraction of cells killed i.e. integrated area above the response curve, ranging from 0 = all cells surviving at the highest dose, to 10 = 100% kill at the lowest dose). Data are shown as median-based box plots (**B**) or dot plots with mean and SD (**C**) (n ≥ 5). Classification of cell lines is indicated by dashed lines. H&N, Head and neck; NSCLC, non-small cell lung cancer; SCLC, small cell lung cancer.

TRX-E-009-1 (Fig. 1A) is a third-generation BP structurally related to TRX-E-002-1^18^. TRX-E-009-1 showed activity against a broad range of cancer types. Of the 240 cell lines assessed in the Eurofin’s Oncopanel, 10 cell lines failed to achieve an IC_50_ below the highest concentration tested (30 μM), of the remaining 230 cell lines, the average IC_50_ was 0.428 μM. Only those cancer types represented by five or more cancer cell lines are shown (Fig. 1B). TRX-E-009-1 demonstrated cytotoxic activity against all cancer types but was broadly, and strongly, active across a panel of kidney, leukaemia, neuroblastoma, osteosarcoma, lymphoma, and melanoma cancer cell lines. In contrast, pancreatic and colon cancer cell lines were less sensitive to TRX-E-009-1.

Reduced sensitivity to TRX-E-009-1 was defined by a percent cell inhibition above 50% at the maximum concentration tested (i.e. at least 50% of the cell population persisted even at the maximum TRX-E-009-1 concentration tested) and/or an IC_50_ above 2 μM. Cell count activity area (CCAA) is a measure which incorporates both percent kill and IC_50_, and provides a clearer indication of sensitivity/insensitivity profiles than IC_50_ alone. Cancer cell lines considered highly sensitive to TRX-E-009-1 had CCAA lower quartile values above 4.5 and included kidney, lymphoma, neuroblastoma, osteosarcoma, and sarcoma cell lines (Fig. 1C). In addition, bladder, head and neck, leukaemia, liver, non-small cell lung cancer (NSCLC), prostate, and small cell lung cancer (SCLC) cell lines all had CCAA median values either above 4.5 or bordering 4.5 and a lower quartile above 3.5 (considered an “insensitive” response). Only colon and pancreatic cancer had median values bordering the insensitive range, suggesting these cancer types may not be suitable targets for clinical translation of TRX-E-009-1. Overall, the majority (~80%) of cell lines assessed in the Oncopanel240 were considered sensitive to TRX-E-009-1, with only 52 cell lines having a CCAA of less than 3.5 (Fig. 1C) and only 24 cell lines (10%) having an IC50 >1 μM.

The induction of cleaved (activated) caspase 3/7 in (CC3/7) response to TRX-E-009-1 was also assessed across the Oncopanel. Analysis of the data indicated a strong correlation between the induction of cleaved caspase 3/7 at a low TRX-E-009-1 concentration and TRX-E-009-1 cytotoxic activity (5 fold CC3/7 level against CCAA: R = −0.7115, P < 0.0001, Supplementary Fig. S1A). Additionally, the maximum level of CC3/7 detected correlated with TRX-E-009-1 sensitivity (maximum CC3/7 level against CCAA: R = 0.5665, P < 0.0001, Supplementary Fig. S1B).

### TRX-E-009-1 and 002-1 Show Similar Potency in Melanoma Models

**Figure 2:**
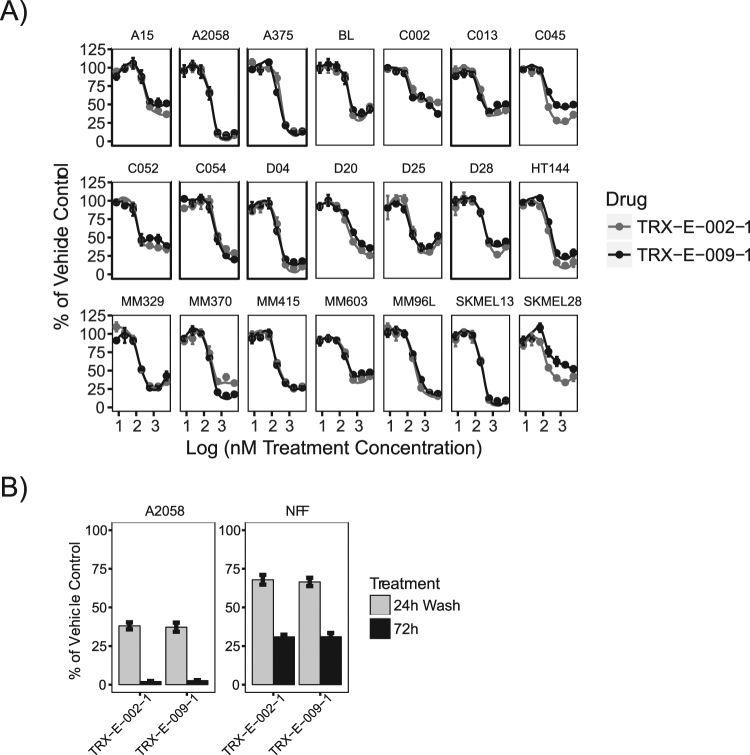
(**A**) Dose response curves (log best fit) for 21 melanoma tumour sphere cell lines for TRX-E-009-1 and TRX-E-002-1. Points represent mean Cell Titre Glo luminescence (n = 4) normalized to vehicle control (error bars are SD) (**B**). Reversible effects of TRX-E-002-1 and TRX-E-009-1 (both at 300 nM) shown in comparison between continuous exposure 72 h or 24 h exposure followed by a wash (A2058 melanoma cell line and normal control NFF (neonatal fibroblasts). Bars represent mean (n = 8, error bars = SD) resazurin fluorescence normalized to vehicle control.

Melanoma cell lines showed a broad range of sensitivity to TRX-E-009-1 (Fig. 1B), providing a good model to further investigate the molecular basis of sensitivity and mechanism of action. A second independent panel of melanoma cell lines grown as tumour spheres was assessed for sensitivity to both TRX-E-009-1 and TRX-E-002-1. Of the 21 lines investigated, the majority had an IC_50_ of <500 nM (Fig. 2A; Supplementary Table S1). As found with the Oncopanel melanoma cell lines, a subset (one third) of the cell lines were sensitive to killing by these BP compounds, with <20% viability at the highest concentration tested. These lines will be referred to as sensitive. The cell lines with >25% viability at the highest dose appear comparatively resistant to killing by the compounds and are referred to as resistant. Subsequent experiments utilized A2058, D04, SKMEL13, and A375 as representative of sensitive cell lines, while A15, SKMEL28, C013, and C002 were used to assess the effects of TRX-E-009-1 and TRX-E-002-1 on resistant lines.

The effects of TRX-E-002-1 were also investigated against adult melanocytes, neonatal melanoblasts and fibroblasts (Supplementary Fig. S2). Normal cells had IC_50_ values of ~50 to 300 nM, similar to the melanomas, but retained >25% viability at the highest doses. Treatment with the compounds was reversible, both sensitive melanoma (A2058) and resistant normal cells (NFF) were able to recover following the removal of either compound after 24 hours of treatment (Fig. 2B).

### Third-generation BP Compounds Cause Mitotic Arrest

**Figure 3:**
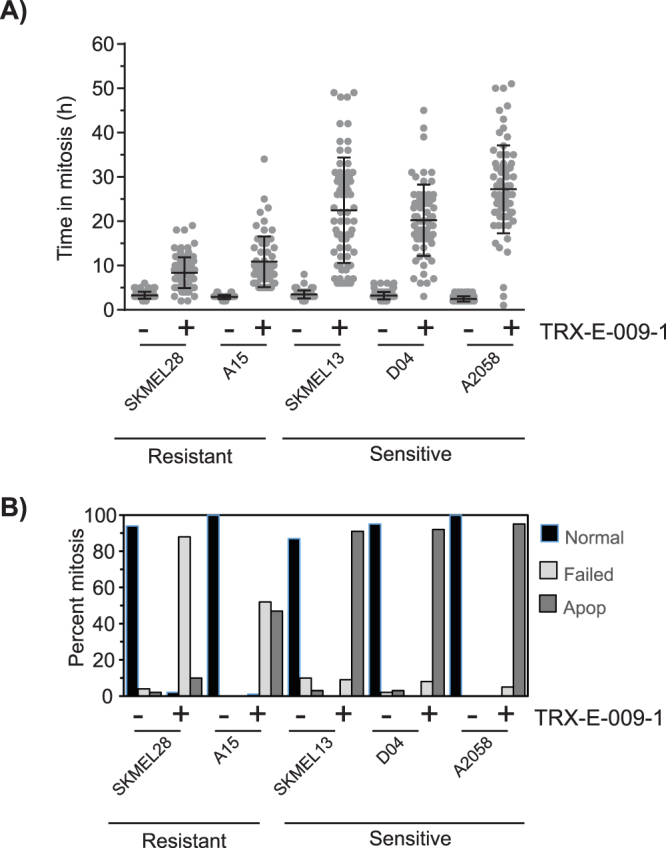
Mitotic delay in melanoma cell lines treated with 300 nM TRX-E-009-1 by analysis of time-lapse microscopy images. (**A**) Time spent in mitosis by each cell is extended in response to treatment, and the delay is longer in the sensitive melanoma lines compared to the resistant lines (≥150 cells quantitated per treatment). (**B**) Proportion of mitotic cells exhibiting normal or failed mitosis or apoptosis. In sensitive cell lines ≥90% of cells undergo apoptosis after mitosis when treated, while the resistant lines predominately fail mitosis without undergoing apoptosis.

In an effort to define the molecular target and mechanism of action of TRX-E-009-1, time-lapse microscopy of treated cells was performed over a three-day period. Cells were treated with 300 nM and 3 μM TRX-E-009-1 to encompass the IC_50_ range of both sensitive and resistant lines. Both concentrations had similar effects and the data from the 300 nM treatments is shown. TRX-E-009-1 delayed all cells in mitosis (Fig. 3A) however, there was a marked difference in the duration of the mitotic arrest between the sensitive and resistant lines, with the sensitive lines having a longer mitotic arrest. This delay was confirmed by immunostaining of cells treated for 24 h with TRX-E-009-1. In treated wells, few cells remained attached to the plate. Detached cells had condensed DNA and were strongly stained for the mitotic marker phospho-Histone 3 Ser10 (pH3), the few remaining attached cells were pH3-negative (Supplementary Fig. S3A). Immunoblotting of similarly treated samples revealed sensitive lines had a strong accumulation of pMEK-T286 levels, a marker of Cyclin B/CDK1 activity and mitosis, compared to the low accumulation in the resistant lines (Supplementary Fig. S3B). Nocodazole, a tubulin polymerization inhibitor that also triggers a mitotic arrest, produced a similar mitotic accumulation to TRX-E-009-1 treatment in sensitive and resistant cell lines.

Differences between the sensitive and resistant lines could also be seen in the fate of cells that failed mitosis. Sensitive lines underwent apoptosis during or immediately after the failed mitosis, whereas resistant lines failed mitosis without undergoing apoptosis (Fig. 3B; Supplementary Fig. S4). The sensitive lines also had increased PARP cleavage after 48 hours treatment with either TRX-E-009-1 or Nocodazole, indicating these treatments triggered apoptosis, whereas there was little evidence of apoptosis in the resistant lines with either agent (Supplementary Fig. S3B). Phosphorylation of c-JUN was increased rapidly with treatment in TRX-E-009-1 sensitive renal cell carcinoma (RCC) and NSCLC cell lines (Supplementary Fig. S5A), confirming previously published results for TRX-E-002-1 in an ovarian cancer model^16^. Increased JNK activation and c-JUN phosphorylation were also observed in human PBMCs treated *in vitro* with TRX-E-002-1 (Supplementary Fig. S5B,C).

### Third-generation BP Compounds Inhibit Tubulin Polymerization

**Figure 4:**
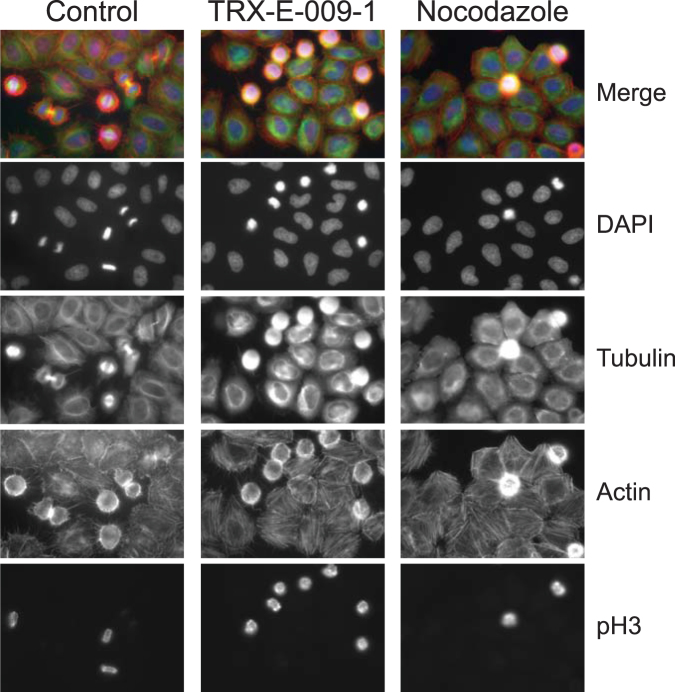
TRX-E-009-1 treatment disrupts microtubule networks. Immunofluorescence staining of Hela cells treated for 4 hours with vehicle control or 300 nM TRX-E-009-1 or Nocodazole (positive control). Stains are as indicated, DNA by DAPI, microtubules by α-tubulin, actin by TRITC-conjugated phalloidin, and mitotic cells by phospho-Histone 3 (Serine 10). Images are 40× magnification, fields measure 222.08 μm × 166.4 μm.

The observed mitotic arrest, and the similarity to Nocodazole effects, suggest these third-generation BPs may also interfere with microtubule structures. TRX-E-009-1 and TRX-E-002-1 had a similar effect on cell viability and potency as Nocodazole, whereas the inactive enantiomer of TRX-E-009-1, TRX-E-009-2, was confirmed as lacking a cytotoxic effect at these concentrations (Supplementary Fig. S6A,B). The microtubule network of control cells was replaced by diffuse staining of tubulin after 24 hours of TRX-E-009-1 treatment (Supplementary Fig. 3A). This observation was further supported by immunofluorescence staining of HeLa cells treated for 4 hours with TRX-E-009-1 (Fig. 4). Staining for α-tubulin showed a loss of microtubule networks in interphase cells and disruption of spindle structures in mitotic cells. Further, reorganization of actin filaments to increased stress fibre formation was shown by phalloidin staining. These results mimic those observed with Nocodazole (Fig. 4). The impact of TRX-E-009-1 on the microtubule structures was similar between resistant and sensitive cell lines, indicating that sensitivity was not a consequence of lack of effect on microtubules (Supplementary Fig. S7, 8).

**Figure 5:**
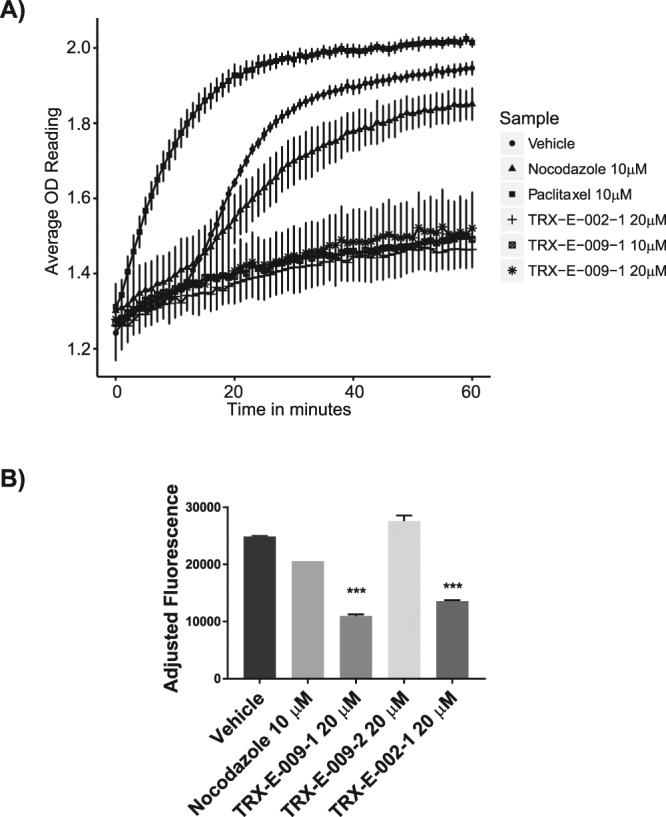
TRX-E-002-1 and TRX-E-009-1 inhibit tubulin polymerization in a cell free assay more potently than the Nocodazole control (**A**). Polymerized tubulin optical density over time (mean +/− standard deviation, n = 3) (**B**). Reduction in fluorescence of the Colchicine-tubulin complex by the active compounds TRX-E-009-1 and TRX-E-002-1 but not the inactive TRX-E-009-2 indicates competitive binding of Colchicine. Bars represent mean adjusted fluorescence readings obtained by subtracting the fluorescence of each corresponding non-Colchicine containing control from the treatment complexes indicated (i.e. [tubulin + Colchicine + drug] – [tubulin + drug], where drug is indicated on the x-axis) +/− range n = 2 (***P < 0.005, as assessed by One-way ANOVA with a Tukey’s multiple comparison test using GraphPad Prism Version 7.03).

The ability of TRX-E-009-1 and TRX-E-002-1 to directly inhibit tubulin polymerization was assessed using an *in vitro* assay. Both of these compounds inhibited tubulin polymerization more effectively than the Nocodazole positive control (Fig. 5A). The effect on polymerization was concentration dependent (Supplementary Fig. S9A,B). A variation of the tubulin polymerization assay was used to assess the competitive binding of TRX-E-009-1 and TRX-E-002-1 against Colchicine to the Colchicine-binding pocket^11^. The two active enantiomers TRX-E-009-1 and TRX-E-002-1, but not the inactive TRX-E-009-2, greatly reduced the fluorescence of the Colchicine-tubulin complex indicating strong competitive binding to the Colchicine binding site on tubulin (Fig. 5B).

### TRX-E-009-1 Significantly Reduces Tumour Volume *in vivo*

**Figure 6:**
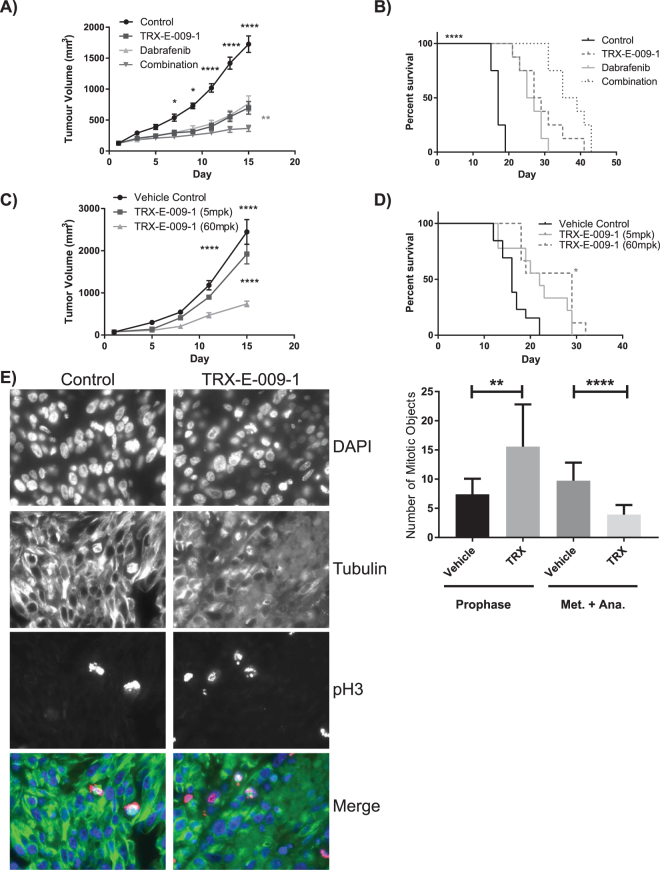
TRX-E-009-1 significantly reduces tumour volume *in vivo*. Tumour size (mean +/− SEM, n = 8) of xenografts (**A**) and survival (assessed by Mantel-Cox Log-rank test) of the inoculated mice (**B**) treated with TRX-E-009-1 or Dabrafenib alone and in combination. Treatment was daily for 15 days. Animals were sacrificed when tumours reached 2000 mm^3^ or morbidity required. Median survival for control animals was 17 days compared to 28 days (TRX-E-009-1), 26 days (Dabrafenib), and 37 days (combination). Growth of a B16F10 melanoma model in immune competent mice (C57/BL6) was significantly inhibited (**C**) and animal survival improved (**D**) by TRX-E-009-1 (n = 8). Mice were treated daily for 15 days by IV with vehicle control, or 5 mg/kg or 60 mg/kg TRX-E-009-1. Staining of tumour sections illustrates a loss of long defined microtubule filaments and mitotic spindles (**E**) as well as an accumulation of cells in prophase (**F**). FFPE sections of A375 xenografts treated daily for 5 days with 80 mg/kg TRX-E-009-1 were immunostained for microtubules (α-tubulin) and mitotic cells (pH3). Images are 63 × magnification, fields measure 138.8 μm × 104 μm. (**F**) mitotic phase objects (prophase or metaphase and/or anaphase) were quantitated from pH3 staining on 20 × magnification images for five fields per tumour for 3 tumours per treatment, bars represent means and SD (assessed by unpaired t-tests with Welch’s correction). (*P < 0.05, **P < 0.01, ***P < 0.005, ****P < 0.0001).

The efficacy of TRX-E-009-1 (solubilized in solutol), alone or in combination with Dabrafenib was investigated in a subcutaneous mouse xenograft model of BRAF mutant (BRAF^V600E^) melanoma (A375). Once tumour volumes reached ~130 mm^3^, mice were assigned into control or treatment groups. Intravenous administration of TRX-E-009-1 (60 mg/kg) and oral administration of Dabrafenib (30 mg/kg) resulted in a tumour growth inhibition compared to control of 61 and 57%, respectively. Administration of TRX-E-009-1 and Dabrafenib in combination resulted in a stronger inhibition of tumour growth (87% compared to vehicle control) (Fig. 6A). Administration of these compounds also resulted in a significant survival advantage, with the combination dosing demonstrating improved activity compared to individual agents alone (Fig. 6B). All treatment groups appeared to be well tolerated as none showed significant loss of body weight over the treatment period (Supplementary Fig. S10A).

A syngeneic mouse melanoma B16F10 model was also used to investigate the anti-cancer activity of TRX-E-009-1 *in vivo*. Treatments were initiated when the average tumour volume reached ~75 mm^3^. Tumour growth inhibition compared to control was 22% and 72% for TRX-E-009-1 at 5 mg/kg and 60 mg/kg, respectively (Fig. 6C). Furthermore, TRX-E-009-1 at 60 mg/kg significantly improved survival compared to control (Fig. 6D). In this more aggressive model, animals lost weight when treated with TRX-E-009-1 at 60 mg/kg but this was not sufficient to require sacrifice (Supplementary Fig. S10B).

### TRX-E-009-1 Disrupts Microtubules *in vivo*

The effects of TRX-E-009-1 on the tumour microtubule network were similar to the effects observed *in vitro*. A375 tumours were implanted as described previously and treated for 5 days (TRX-E-009-1 80 mg/kg IV daily or vehicle control) prior to harvesting. TRX-E-009-1 treatment resulted in a loss of the long microtubule filaments in favour of shorter filaments and dispersed tubulin staining. This was also evident in the mitotic spindles which were less robust than in the controls (Fig. 6E). Quantitation of the effects of TRX-E-009-1 on mitosis revealed there was a significant increase in the proportion of prophase cells and a reduction in metaphase and anaphase cells in the treated tumours (Fig. 6F). This reflects the defective microtubule spindles and the prophase accumulation observed in *in vitro* treated cells (Supplementary Fig. S3).

## Discussion

We investigated the mechanism of action and anti-cancer activity of third-generation BP compounds, TRX-E-009-1 (Trilexium) and the current clinical candidate TRX-E-002-1 (Cantrixil) (NCT02903771). Both of these compounds show potential as broad acting anti-cancer agents, with TRX-E-002-1 previously shown to be effective against *in vitro* and *in vivo* models of ovarian cancer^16^. Moreover, TRX-E-002-1 was effective against chemoresistant cancer stem cell (CD44+ MyD88+) populations in both 2D and 3D models as well as having potent cytotoxic activity against non-stem like ovarian cancer cells. Further to these findings we have shown that both TRX-E-002-1 and TRX-E-009-1 interfere with microtubule dynamics. Anti-microtubule agents, based on their tubulin binding site and subsequent effect on microtubule dynamics, are generally categorized as either stabilizing such as taxanes, or destabilizing such as Colchicine and the vinca-alkaloid family^19^. Our data indicates potent inhibition of tubulin polymerization by these third generation BPs. Furthermore, their ability to compete with Colchicine for binding indicates they share the same or an overlapping binding site. These data clearly identify tubulin as a molecular target of third-generation BPs both *in vitro* and *in vivo* and this activity likely contributes to their cytotoxic effects.

This finding is supported by previous work showing that ME-344 (NV-128), a second-generation BP, also binds to the Colchicine domain and inhibits microtubule polymerization^11^. This occurred in addition to other mechanisms of action including a significant increase in mitochondrial ROS production^11,13^. Inhibition of either mechanism rescued cells from ME-344-induced cell death to variable extents depending on the cell line. Furthermore, microtubule-interfering drugs commonly activate JNK signalling, leading to phosphorylation of cJUN^20–22^, and inhibiting JNK activity reduces the cytotoxic effect of these drugs. These effects have also been observed in ovarian cancer cells treated with TRX-E-002-1^16^. In this work they demonstrated that levels of p-JNK and p-cJUN increased in response to TRX-E-002-1 and that co-treatment with a JNK inhibitior, as well as siRNA knockdown of the downstream cJUN, resulted in reduced cell death by TRX-E-002-1. Our data illustrates the level of JNK activation is related to the strength of the mitotic arrest triggered by the treatment and that a critical level of JNK activation may be required to result in cell death. Alternatively, it is possible that JNK activation is necessary but not sufficient for TRX-E-009-1 or TRX-E-002-1-induced cell death.

When assessing responses to third-generation BP compounds, comparison of IC_50_ values is insufficient for distinguishing between sensitive and resistant melanoma cell lines; instead, the differences in the outcome of their mitotic arrest must be considered. As with most anti-microtubule agents, cells treated with either TRX-E-009-1 or TRX-E-002-1 arrest in mitosis due to the activation of the spindle assembly checkpoint (SAC), which detects defects in the mitotic spindle function and blocks progression through mitosis by inhibiting the activity of the anaphase-promoting complex/cyclosome (APC/C)^23^. It is the outcome of this mitotic arrest that determines cell fate and subsequent definition as sensitive or resistant to these third-generation BP compounds. The mechanism underlying the difference in the duration of the mitotic arrest is unclear. The ability of the compounds to trigger a mitotic arrest in all cell lines is a clear indication that the SAC is functional, although its ability to maintain a mitotic arrest is impaired in the resistant lines. Sensitive cell lines have a longer mitotic arrest than resistant, which exit mitosis without correcting their defective mitosis, a process which is termed mitotic slippage. The duration of the mitotic arrest is a major factor in determining whether there is mitotic slippage and survival, or apoptosis^24^. An extended delay in mitosis favours apoptosis over slippage^25^, with the balance of the inactivation and destruction of anti-apoptotic BCL-2 family members and the stability of pro-apoptotic BH-3 only proteins NOXA and BIM determining the outcome^26^.

There have been many novel microtubule destabilizing agents that bind at or near the colchicine-binding site on tubulin, with Combrestatin A4 having the most extensive clinical data. Combrestatin A4 has similarities in binding site, potency, and structure to TRX-E-002-1 and TRX-E-009-1. In addition to the anti-microtubule activity thought to be its primary effect on tumours, Combrestatin has vascular disruptive activity^27^, suggesting that the TRX drugs may have similar effects. Combrestatin has little clinical activity as a single agent, but has been reported to have significant activity in combinations with other chemotherapies^28^. TRX-E-009-1 combined strongly with Dabrafenib in melanoma in A375 (*BRAF*^V^^600E^) xenografts, suggesting that the TRX family are also likely to find clinical value in rational combination therapies. In the case of the BRAF inhibitors, these have been shown to increase the phosphorylation of the pro-apoptotic BH-3 only protein BIM, thereby increasing its stability and activity^29–31^. BIM is a known promoter of anti-microtubule agent induced cell killing^26^, thus the combination of these agents should enhance the activity of this critical pro-apoptotic protein enhancing the cell killing and tumour control as observed.

Pharmacology, genotoxicity and toxicology studies demonstrate that TRX-E-002-1 has an acceptable toxicity profile in rats and dogs, with reversible gastrointestinal tract toxicities the primary toxicity^18^. These findings are not unexpected for a drug which targets rapidly dividing cells and are consistent with similarly potent chemotherapeutic agents. TRX-E-002-1 induced irreversible degeneration of spermatogenic elements lining the seminiferous tubules. This unusual toxicity may point to the expression of a unique target for TRX-E-002-1 in male reproductive organs. TRX-E-002-1 was without genotoxicity and cardiotoxicity at clinically relevant concentrations^18^.

Anti-microtubule agents form a critical backbone to current chemotherapy regimens, with both microtubule-stabilizing and destabilizing agents in frequent clinical use. Third-generation BPs have shown promise in pre-clinical models, as described here and elsewhere^16^, and may offer a novel anti-microtubule strategy. Moreover, for reasons not yet clear, these agents appear to have strong activity against normally chemo-resistant cells and stem-like cancer cells purported to be the cause of disease recurrence in many cancer types^16^. Thus, third-generation BPs may be effective in indications or discrete populations of stem-like cancer cells not responsive to currently approved anti-microtubule agents. Currently Cantrixil is under clinical investigation in a Phase I study of ovarian cancer (NVGN-002-101, NCT02903771). Biomarker identification studies to define resistant and sensitive populations prior to treatment initiation could help to determine those patients most likely to receive the greatest clinical benefit in future studies.

## Electronic supplementary material

Supplementary Data
