# Supplementary material for: Mechanism of action of the third generation benzopyrans and evaluation of their broad anti-cancer activity in vitro and in vivo
Source: Sci Rep. 2018 Mar 23;8:5144. doi: 10.1038/s41598-018-22882-w (PMC5865165; doi:10.1038/s41598-018-22882-w)

Mechanism of action of the third generation benzopyrans and evaluation of their broad anti-cancer activity *in vitro* and *in vivo*

Alexander J. Stevenson[1], Eleanor I. Ager[2], Martina A. Proctor[1], Dubravka Škalamera[1], Andrew Heaton[2,3], David Brown[2,3], Brian G. Gabrielli[1*]

Supplementary Material

Supplementary Figure Legends

Supplementary Table 1:

Calculated IC_{50} values for each of the 21 melanoma tumor sphere lines shown in Fig. 2A and their sensitivity classification based on viability at the highest treatment concentration. The inactive enantiomer TRX-E-009-2 is also included where data was available. Cell lines where no IC_50 was found by the highest treatment concentration (5.4 μM) are listed as NA. Calculations were performed in R-Studio (1-5).

Supplementary Figure 1:

Induction of cleaved caspase 3/7 (CC3/7) (5-fold above control) at a low TRX-E-009-1 concentrations strongly correlates with TRX-E-009-1 cytotoxic activity (**A**) while maximum level of CC3/7 detected negatively correlated with TRX-E-009-1 sensitivity (**B**). A Pearson correlation analysis was performed using GraphPad Prism version 6.05 on log-transformed CC3/7 and cell count activity area data from Eurofins Oncopanel 240. Pearson R value = -0.7115 and P < 0.0001 (**A**) and Pearson R value = 0.5665, P < 0.0001 (**B**).

Supplementary Figure 2:

'Normal' cell lines are resistant to treatment with TRX-E-002-1. Dose response experiments were performed for a panel of 'normal' lines; adult human melanocytes (HEMa), and neonatal fibroblasts (NFF) and melanoblasts (QF1610). Cells were treated with 7 point 3-fold dose curve of TRX-E-002-1 for 72 hours (5.4 μM to 7.4 nM), means of resazurin fluorescence are represented as percent of vehicle control for comparison between cell lines, (error bars = SD, n = 4). All 3 lines are classed as resistant as > 25 % viability at the highest treatment concentration.

Supplementary Figure 3:

TRX-E-009-1 treated cells undergo mitotic delay. Immunofluorescence staining of A2058 melanoma cells (**A**) treated with either 300 nM TRX-E-009-1 or vehicle control for 24 hours. Loosely adherent/floating cells were also collected, spun down, and stained (indicated). DNA is stained with DAPI, microtubules with α-tubulin and phospho-Histone 3 (Ser10) was used as a mitotic marker. **B**. Immunoblotting of similarly treated subset of sensitive and resistant melanoma cells (indicated). Cells were treated as in **A** with the addition of 300 nM Nocodazole as a positive control treatment and a 48 hour treatment for cleaved PARP. Antibody stains are as indicated.

Supplementary Figure 4:

Melanoma cells treated with TRX-E-009-1 are delayed in mitosis and depending on sensitivity either undergo apoptosis (**A**) or mitotic slippage (**B**). Cells were treated with 300 nM TRX-E-009-1 or vehicle control for 72 hours, images were captured every 30 minutes via time-lapse microscopy. Selected montages are representative of cell fate post mitotic delay, frame numbers are indicated at the bottom of each image.

Supplementary Figure 5:

Phosphorylated JNK and cJUN are increased by TRX-E-009-1. **A.** 786-O (renal cell adenocarcinoma) and NCI-H661 (Non-small cell lung cancer) cells treated with indicated concentrations and lengths of TRX-E-009-1 immunoblotted for p-cJUN and GAPDH (loading control). Human PBMCs treated with indicated concentrations and lengths of TRX-E-002-1 probed for p-cJUN (**B**) and pJNK (**C**).

Supplementary Figure 6:

Melanoma cell lines have similar responses to TRX-E-009-1 treatment as to Nocodazole. Treatment of melanoma cell lines with a 7 point 3-fold dose curve of the indicated drugs (TRX-

E-009-2 is the inactive enantiomer of TRX-E-009-1) for 72 hours. Data is mean of Cell Titre Glo luminescence (**A**) or resazurin fluorescence (**B**) represented as percent of vehicle control for comparison between cell lines (error bars = SD, n = 4). Experiments were performed on 3D tumor sphere cultures (**A**) and traditional 2-dimensional culture (**B**).

Supplementary Figure 7:

TRX-E-009-1 treatment disrupts microtubule network in resistant cell lines. Immunofluorescence staining of a panel of resistant melanoma cell lines treated with 300 nM TRX-E-009-1 or vehicle control for 24 hours. Images are 63x magnification, fields measure 138.8 μm x 104 μm.

Supplementary Figure 8:

TRX-E-009-1 treatment disrupts microtubule network in resistant cell lines. Immunofluorescence staining of a panel of sensitive melanoma cell lines treated with 300 nM TRX-E-009-1 or vehicle control for 24 hours. Images are 63x magnification, fields measure 138.8 μm x 104 μm.

Supplementary Figure 9:

Inhibition of tubulin polymerization with TRX-E-009-1 and TRX-E-002-1 is dose-dependent. Cell free tubulin assays as in Fig. 5A with decreasing doses of TRX-E-002-1 (**A**) or TRX-E-009-1 (**B**) along with polymerization inhibition (Nocodazole) or enhancing (Paclitaxel) controls. Data is mean of polymerized tubulin optical density over time (error bars = SD, n = 3).

Supplementary Figure 10:

Treatments of mice in xenograft models did not adversely affect body weight. Athymic (**A**) and C57/BL6 (**B**) mice from Fig. 6 were treated with the indicated drug for 15 days (dotted

line) and weighed every second day. Data is mean weight for each treatment group (error bars = SD, n = 8).

Supplementary Materials and Methods

Primary human melanoblasts, QF1610, were provided by Professor Richard Sturm and cultured as described in (6). Samples were prepared and immunoblotting was performed as previously described in (7).

1. R Core Team (2016) R: A language and environment for statistical computing (R Foundation for Statistical Computing, Vienna, Austria).
2. RStudio Team (2016) RStudio: Integrated Development Environment for R (RStudio, Inc., Boston, MA), 1.0.44.
3. Wickham H (2007) Reshaping Data with the reshape Package. *2007* 21(12):20.
4. Wickham H (2009) ggplot2: Elegant Graphics for Data Analysis. (Springer-Verlag, New York).
5. Wickham H, Hester J, & Francois R (2016) readr: Read Tabular Data).
6. Cook AL*, et al.* (2003) Human melanoblasts in culture: expression of BRN2 and synergistic regulation by fibroblast growth factor-2, stem cell factor, and endothelin-3. *The Journal of investigative dermatology* 121(5):1150-1159.
7. Brooks K*, et al.* (2014) Decatenation checkpoint-defective melanomas are dependent on PI3K for survival. *Pigment cell & melanoma research* 27(5):813-821.

Supplementary Table S1

Cell Line	Drug	Log (IC50 nM)	IC50 nM	Classification
A15	TRXE-002-1	2.881	760	Resistant
A15	TRXE-009-1	2.689	489	Resistant
A2058	TRXE-002-1	2.443	277	Sensitive
A2058	TRXE-009-1	2.466	292	Sensitive
A2058	TRXE-009-2	NA	NA	No Effect
A375	TRXE-002-1	2.589	388	Sensitive
A375	TRXE-009-1	2.506	321	Sensitive
A375	TRXE-009-2	NA	NA	No Effect
BL	TRXE-002-1	2.706	508	Resistant
BL	TRXE-009-1	2.532	340	Resistant
C002	TRXE-002-1	NA	NA	Resistant
C002	TRXE-009-1	2.99	977	Resistant
C013	TRXE-002-1	2.646	443	Resistant
C013	TRXE-009-1	2.503	318	Resistant
C045	TRXE-002-1	2.374	237	Resistant
C045	TRXE-009-1	2.664	461	Resistant
C052	TRXE-002-1	2.408	256	Resistant
C052	TRXE-009-1	2.466	292	Resistant
C054	TRXE-002-1	2.981	957	Resistant
C054	TRXE-009-1	2.933	857	Resistant
D04	TRXE-002-1	2.463	290	Sensitive
D04	TRXE-009-1	2.403	253	Sensitive
D20	TRXE-002-1	2.729	536	Resistant
D20	TRXE-009-1	3.045	1109	Resistant
D25	TRXE-002-1	2.506	321	Resistant
D25	TRXE-009-1	3.701	5023	Resistant
D28	TRXE-002-1	2.718	522	Resistant
D28	TRXE-009-1	2.896	787	Resistant
HT144	TRXE-002-1	2.411	258	Sensitive
HT144	TRXE-009-1	2.368	233	Resistant
MM329	TRXE-002-1	2.4	251	Resistant
MM329	TRXE-009-1	2.251	178	Resistant
MM370	TRXE-002-1	2.658	455	Resistant
MM370	TRXE-009-1	2.463	290	Sensitive
MM415	TRXE-002-1	2.523	333	Resistant
MM415	TRXE-009-1	2.509	323	Resistant
MM603	TRXE-002-1	2.666	463	Resistant
MM603	TRXE-009-1	2.454	284	Resistant
MM96L	TRXE-002-1	2.526	336	Sensitive
MM96L	TRXE-009-1	2.449	281	Sensitive
SKMEL13	TRXE-002-1	2.417	261	Sensitive
SKMEL13	TRXE-009-1	2.388	244	Sensitive
SKMEL13	TRXE-009-2	NA	NA	No Effect
SKMEL28	TRXE-002-1	2.503	318	Resistant
SKMEL28	TRXE-009-1	3.661	4581	Resistant

NA means no IC50 found at tested Concentrations (i.e. >5.4µM)

Supplementary Fig. S1

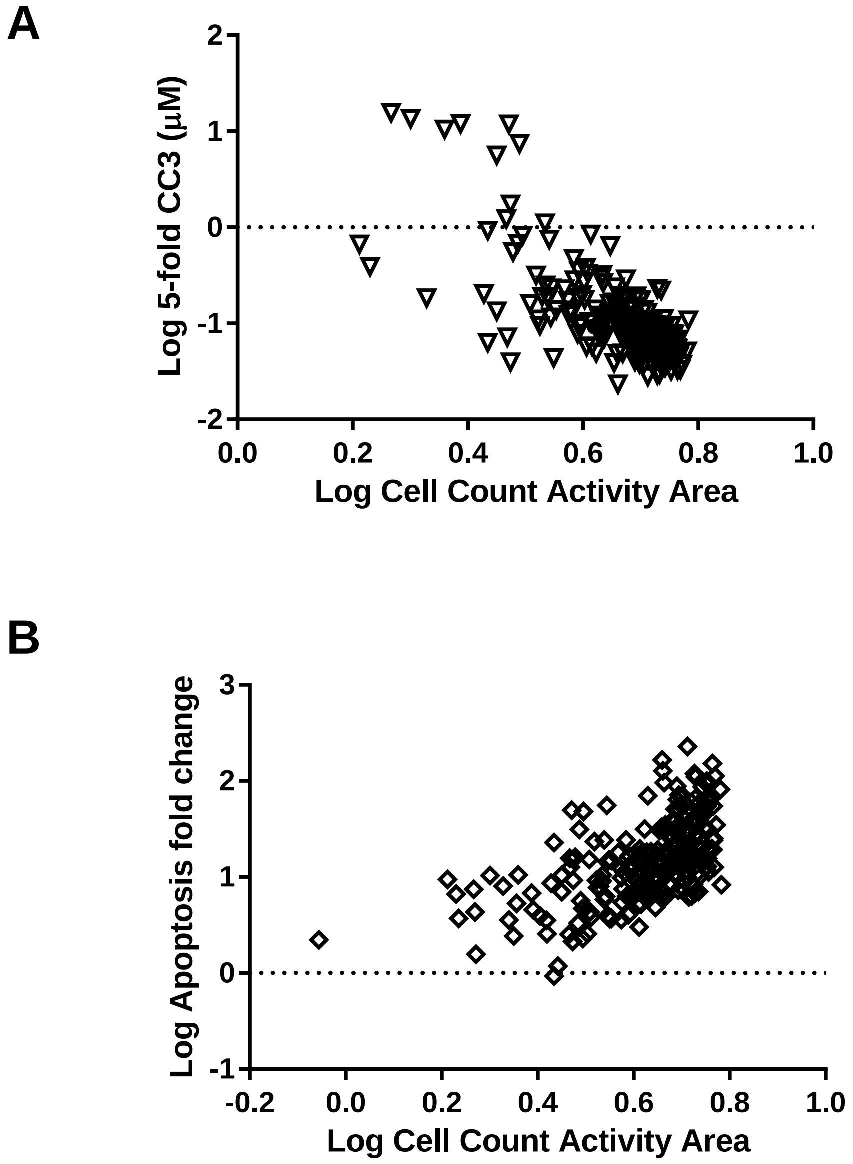

Supplementary Fig. S2

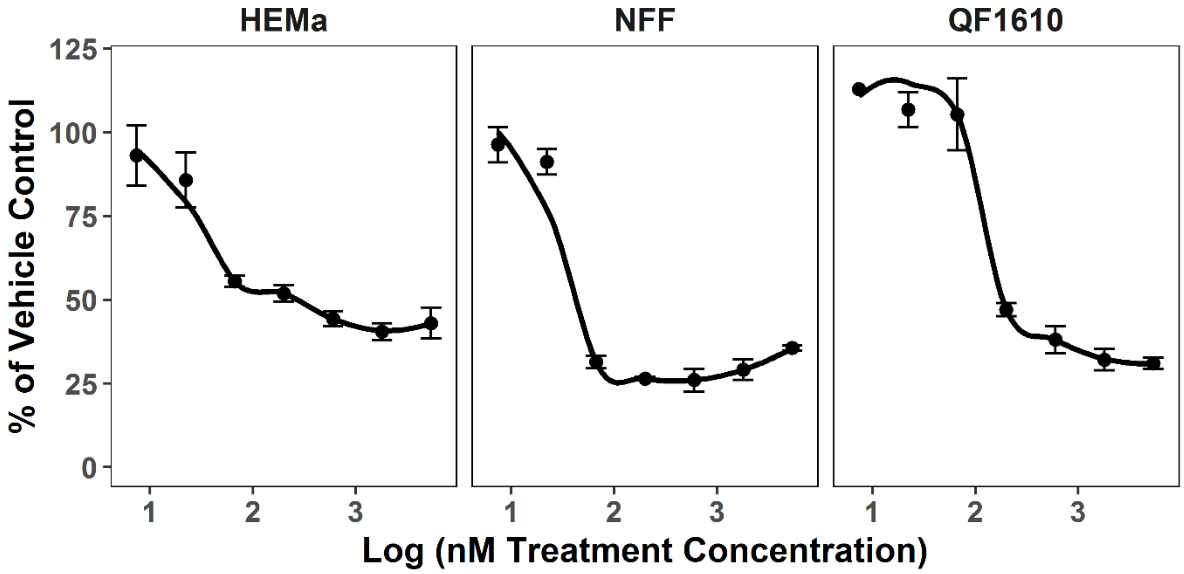

Supplementary Figure S3

A)

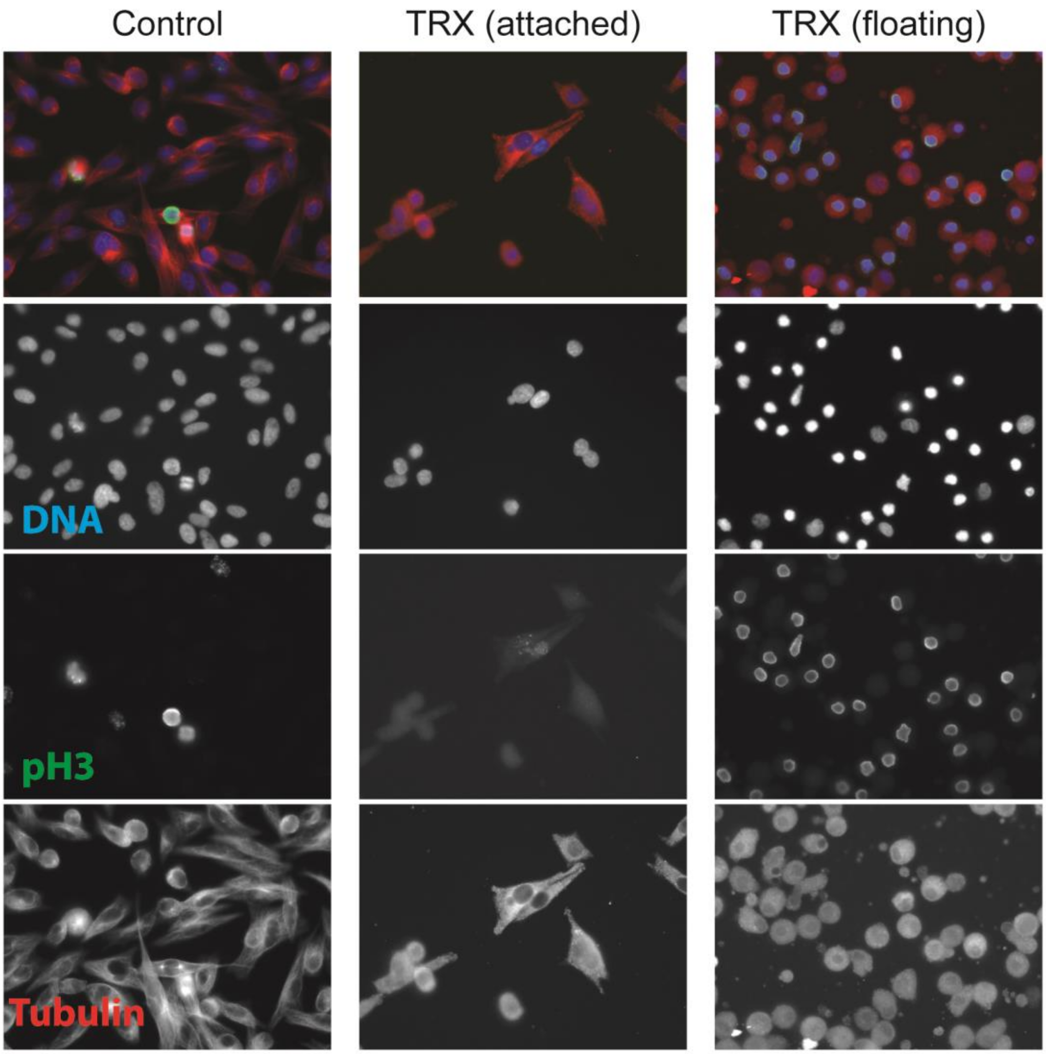

B)

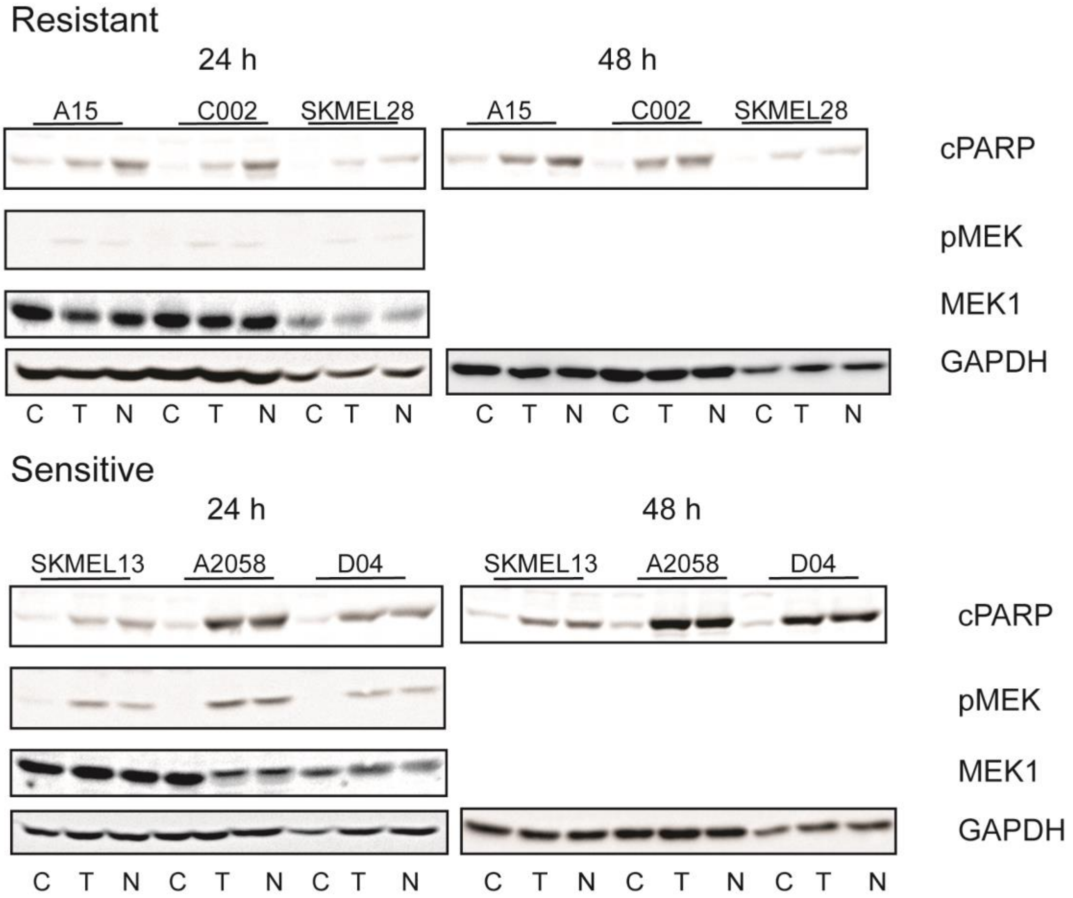

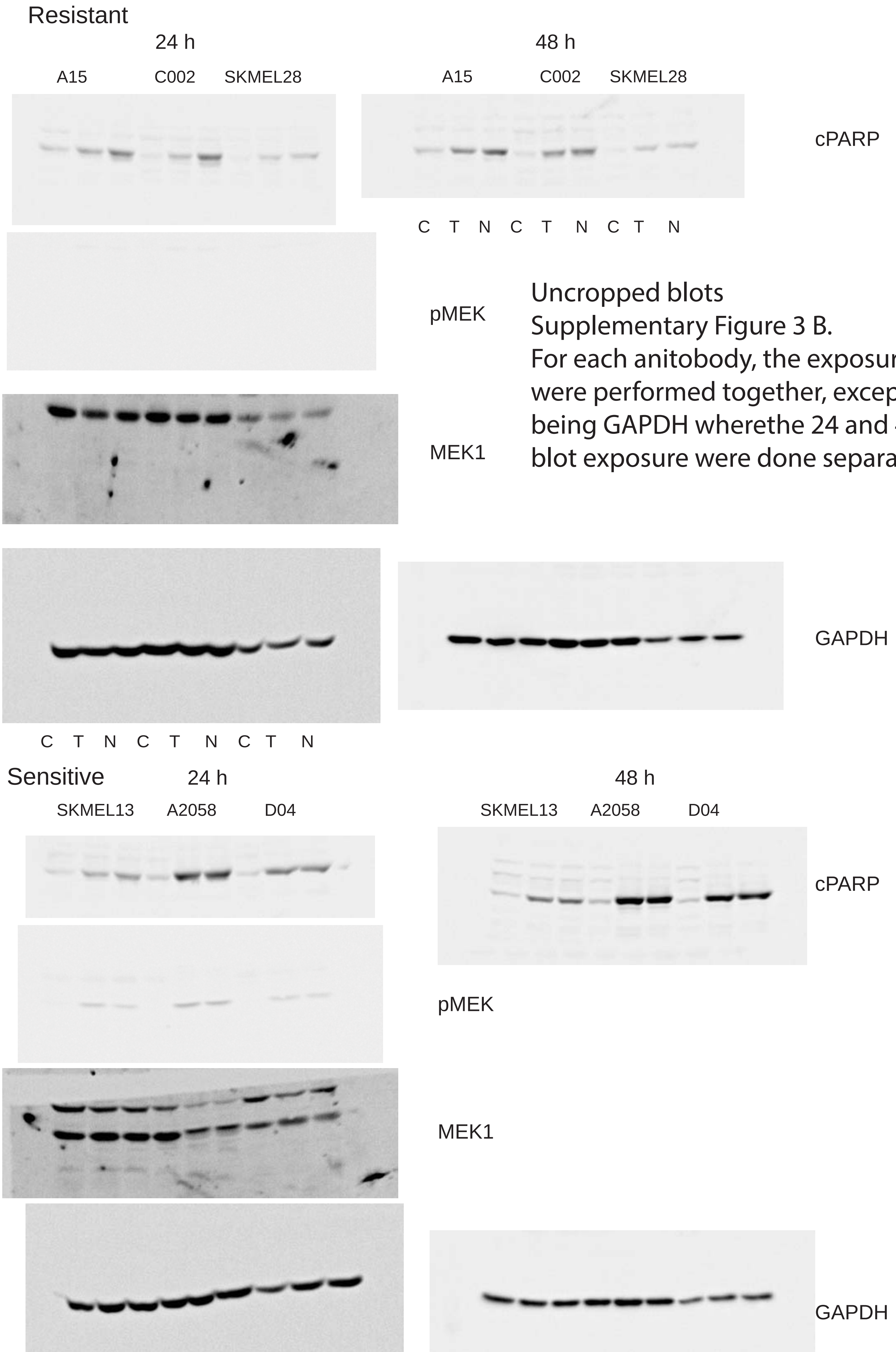

Uncropped blots
Supplementary Figure 3 B.
For each anitobody, the exposure were performed together, exception being GAPDH wherethe 24 and 48 h blot exposure were done separately.

Supplementary Fig. S4A

D04 - Sensitive

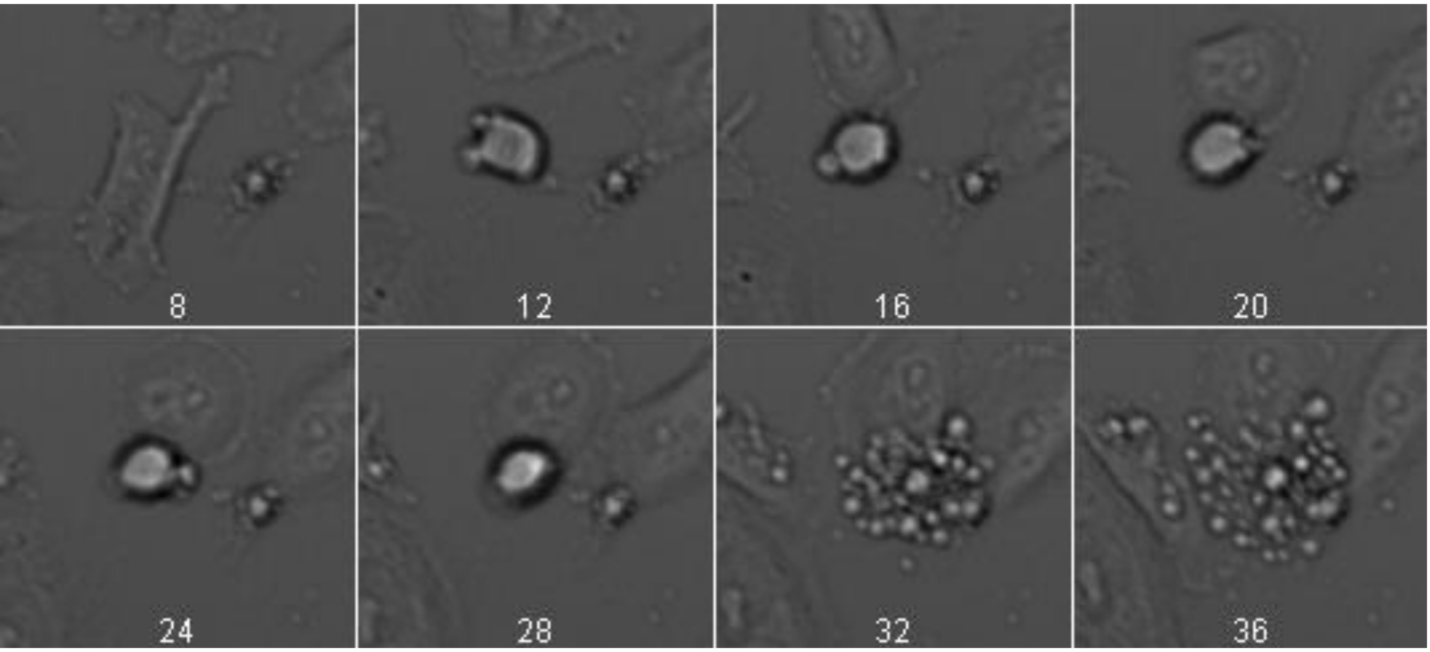

Supplementary Fig. S4B

A15 - Resistant

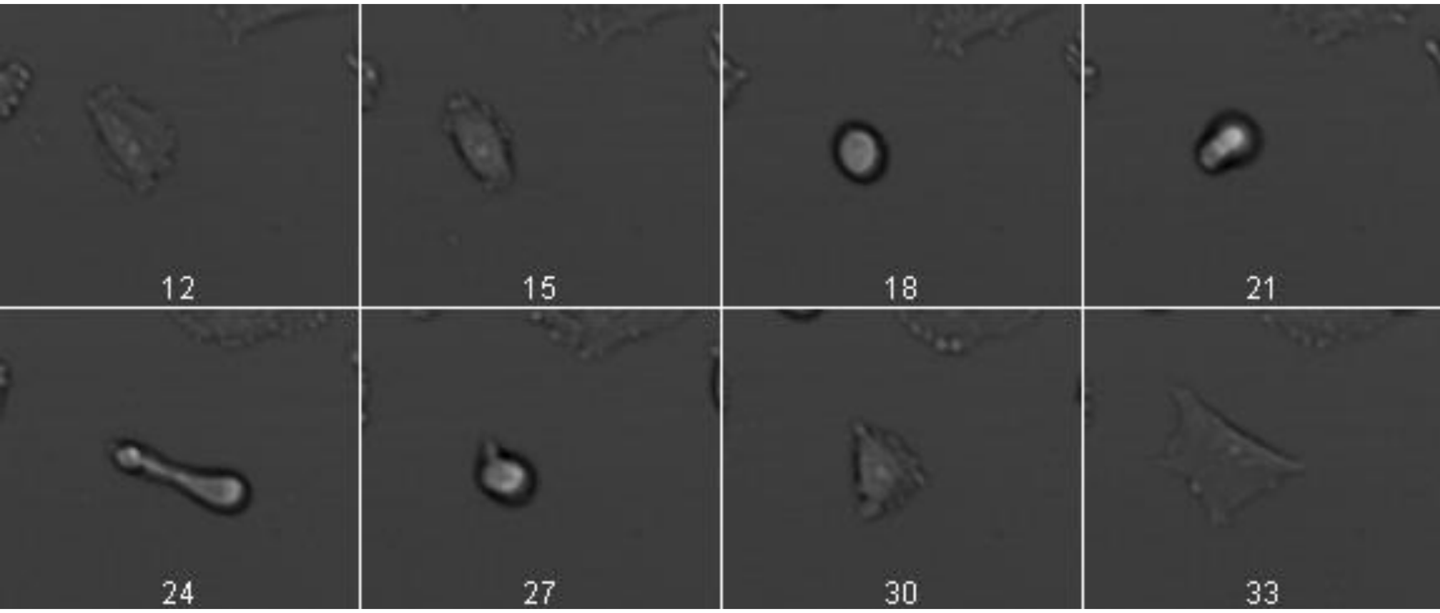

Supplementary Fig. 5A

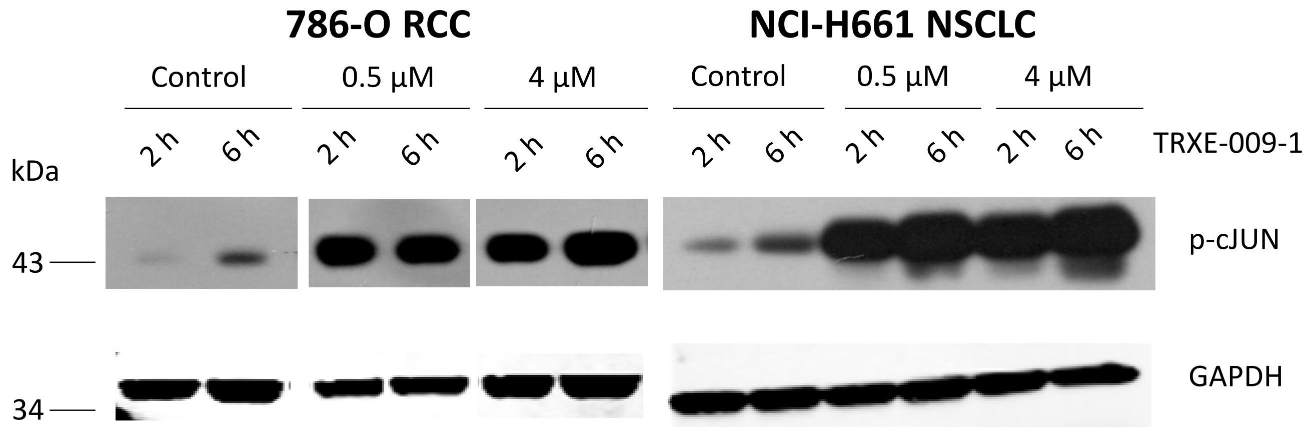

Supplementary Fig. 5B

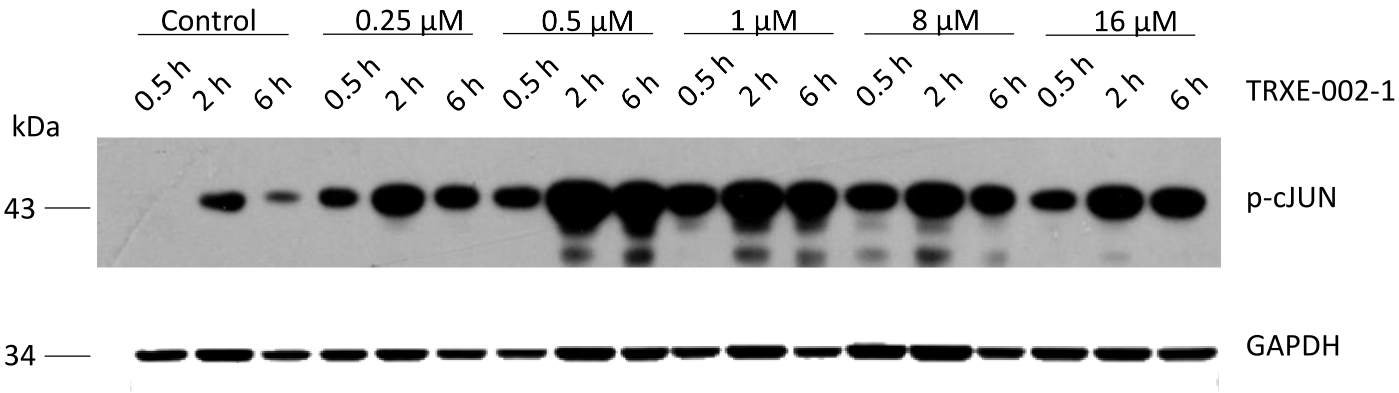

Supplementary Fig. 5C

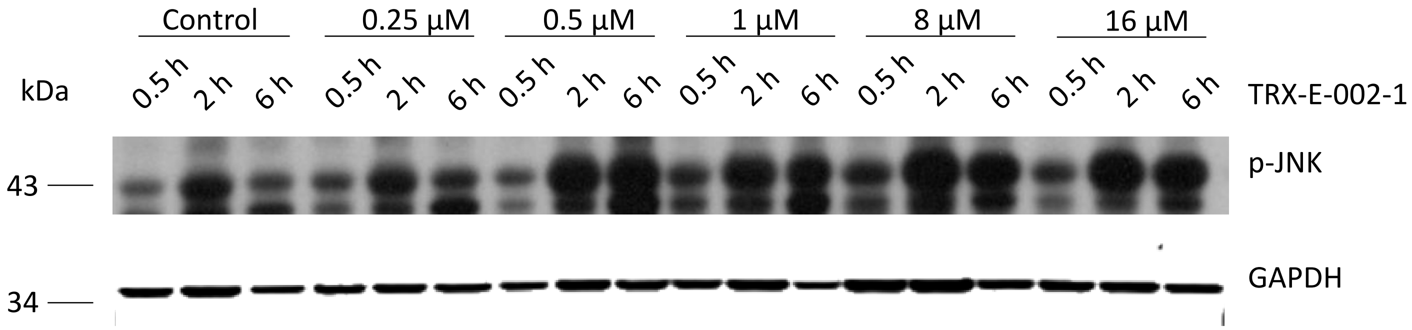

Supplementary Fig. 5A – Uncropped non-greyscale

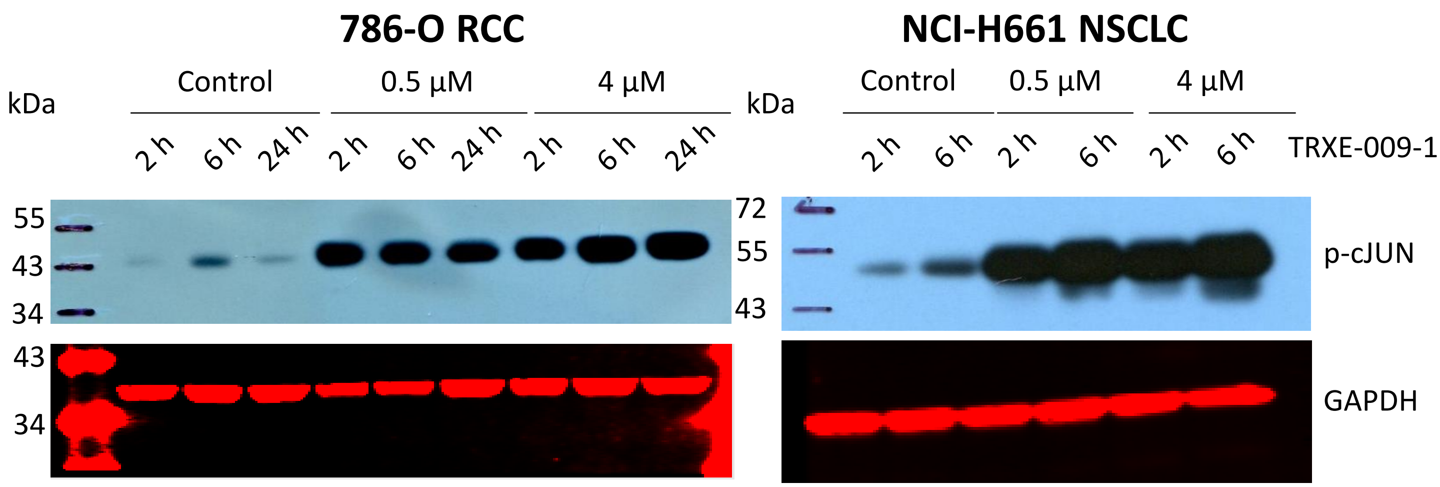

Supplementary Fig. 5B - Uncropped non-greyscale

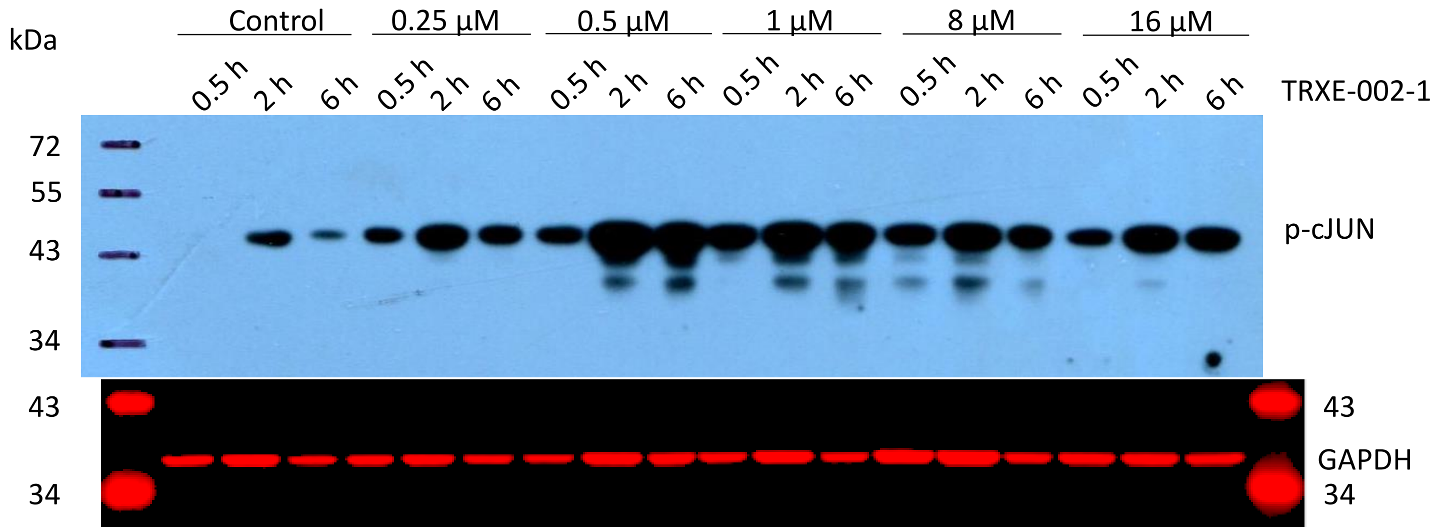

Supplementary Fig. 5C - Uncropped non-greyscale

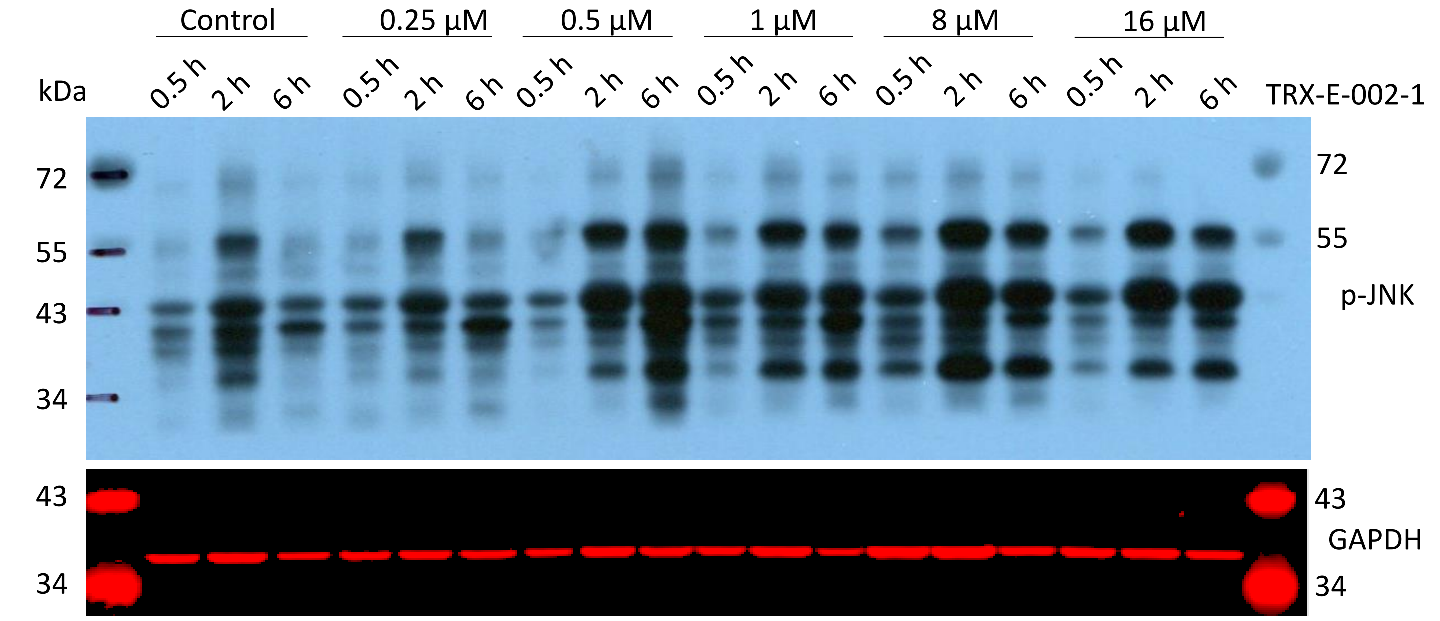

Supplementary Fig. S6A

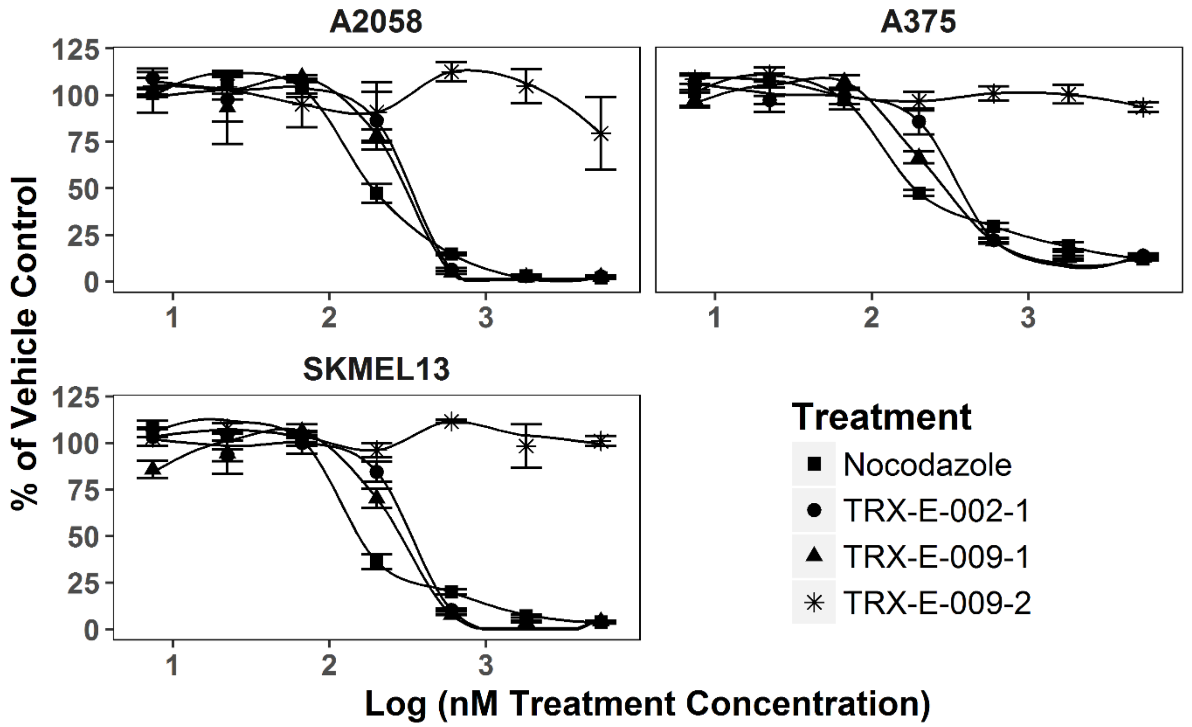

Supplementary Fig. S6B

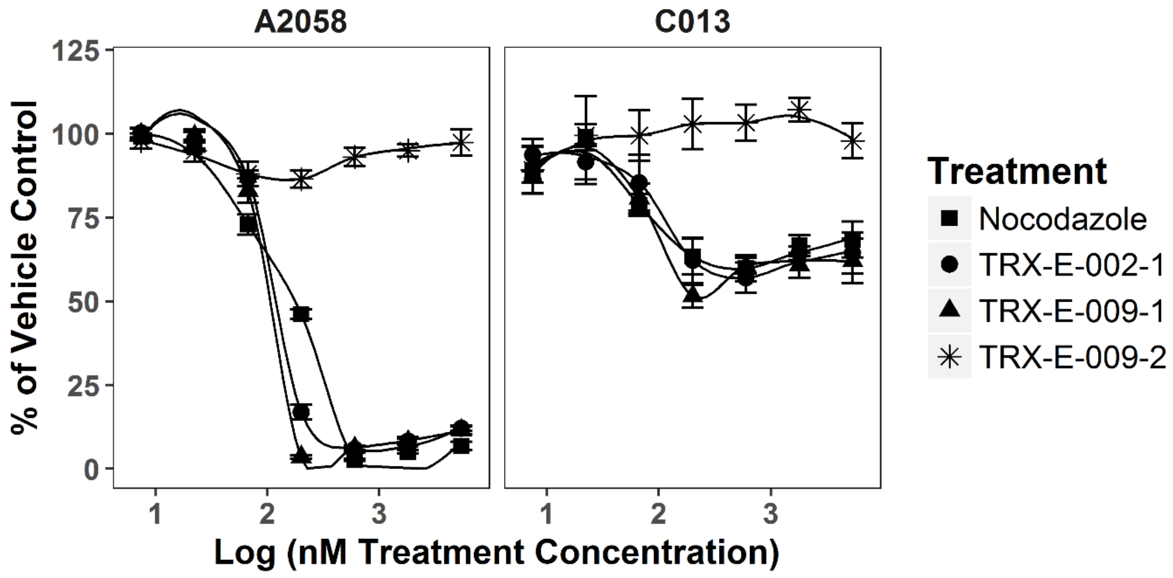

Supplementary Figure S7

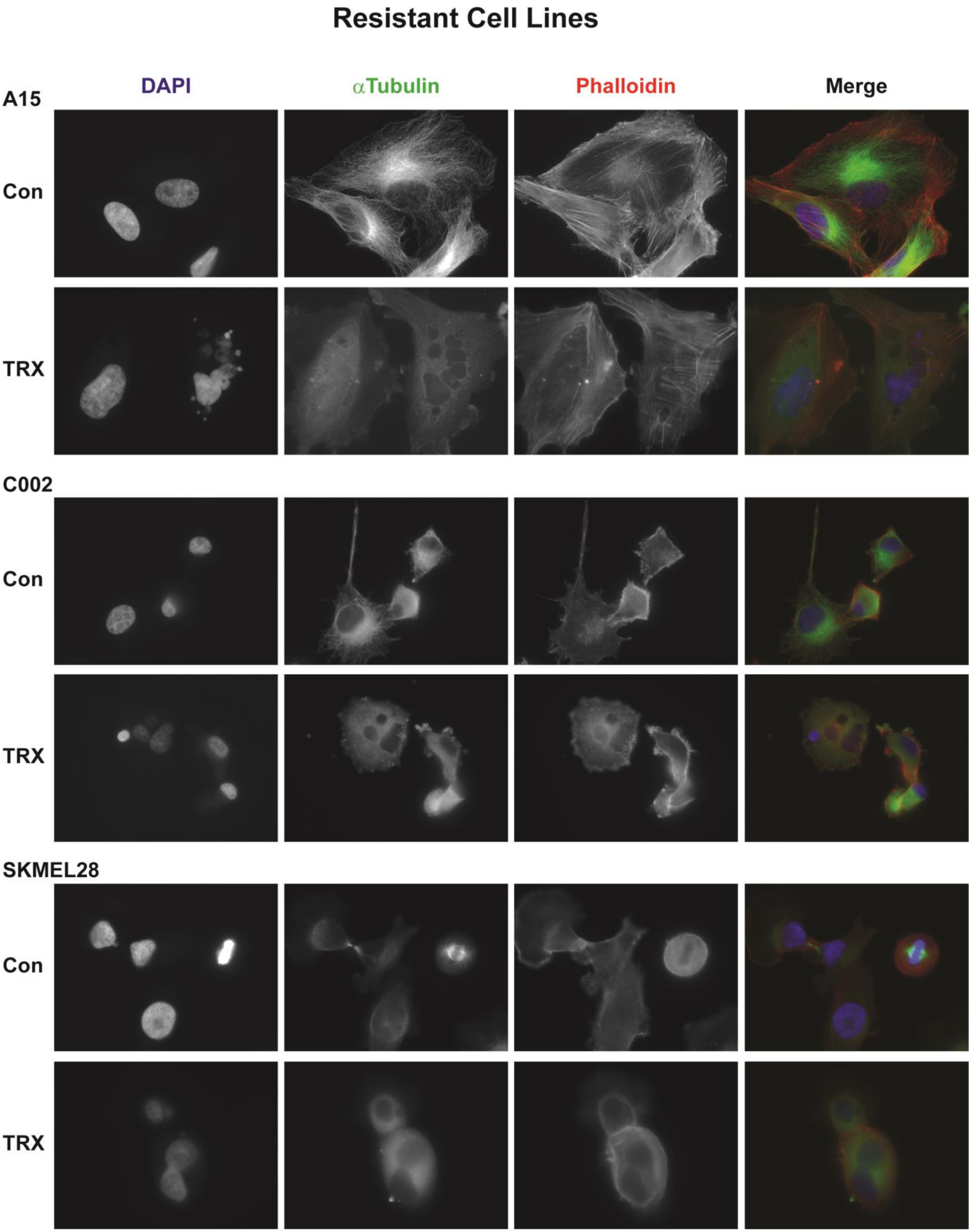

Supplementary Figure S8

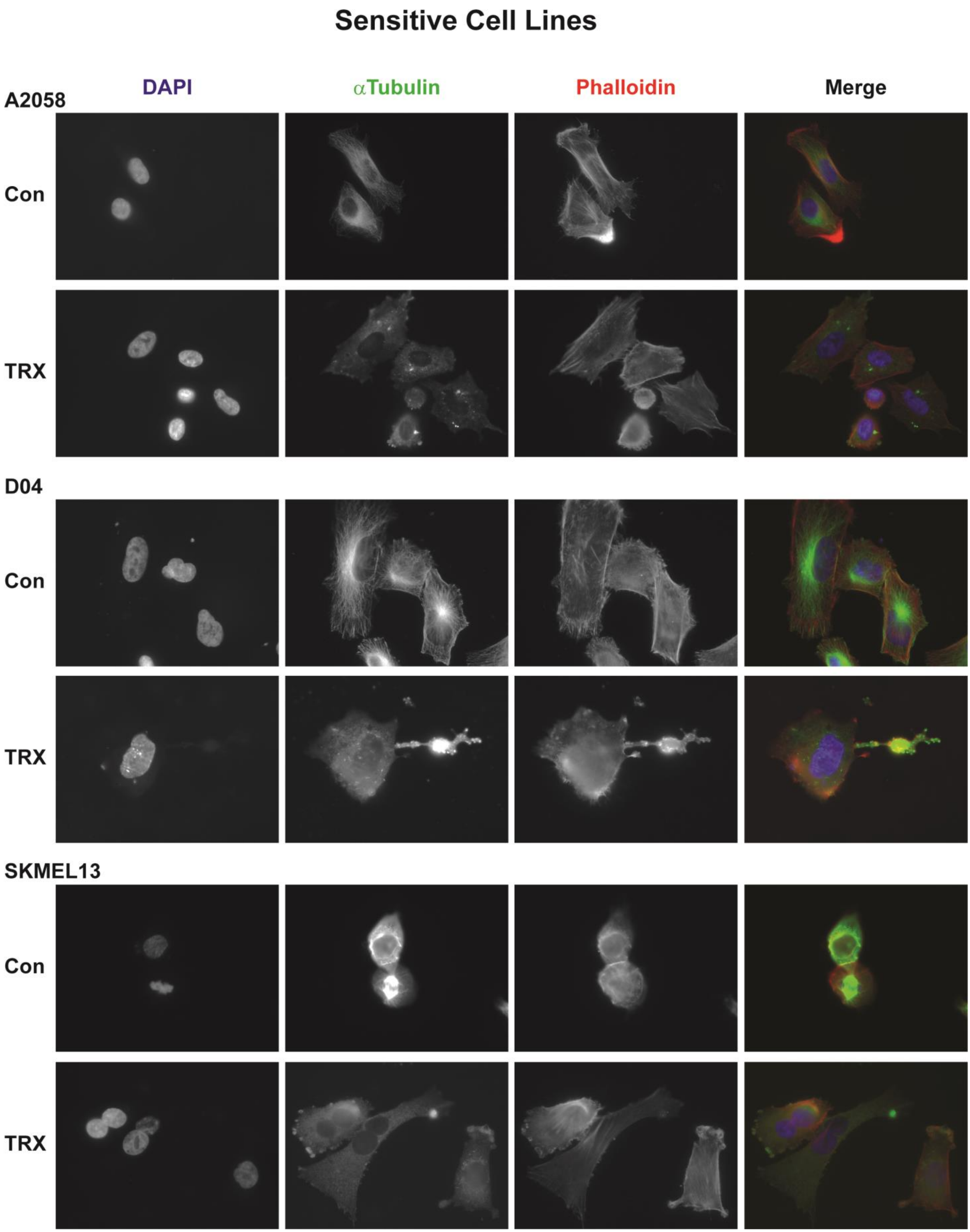

Supplementary Fig. S9A

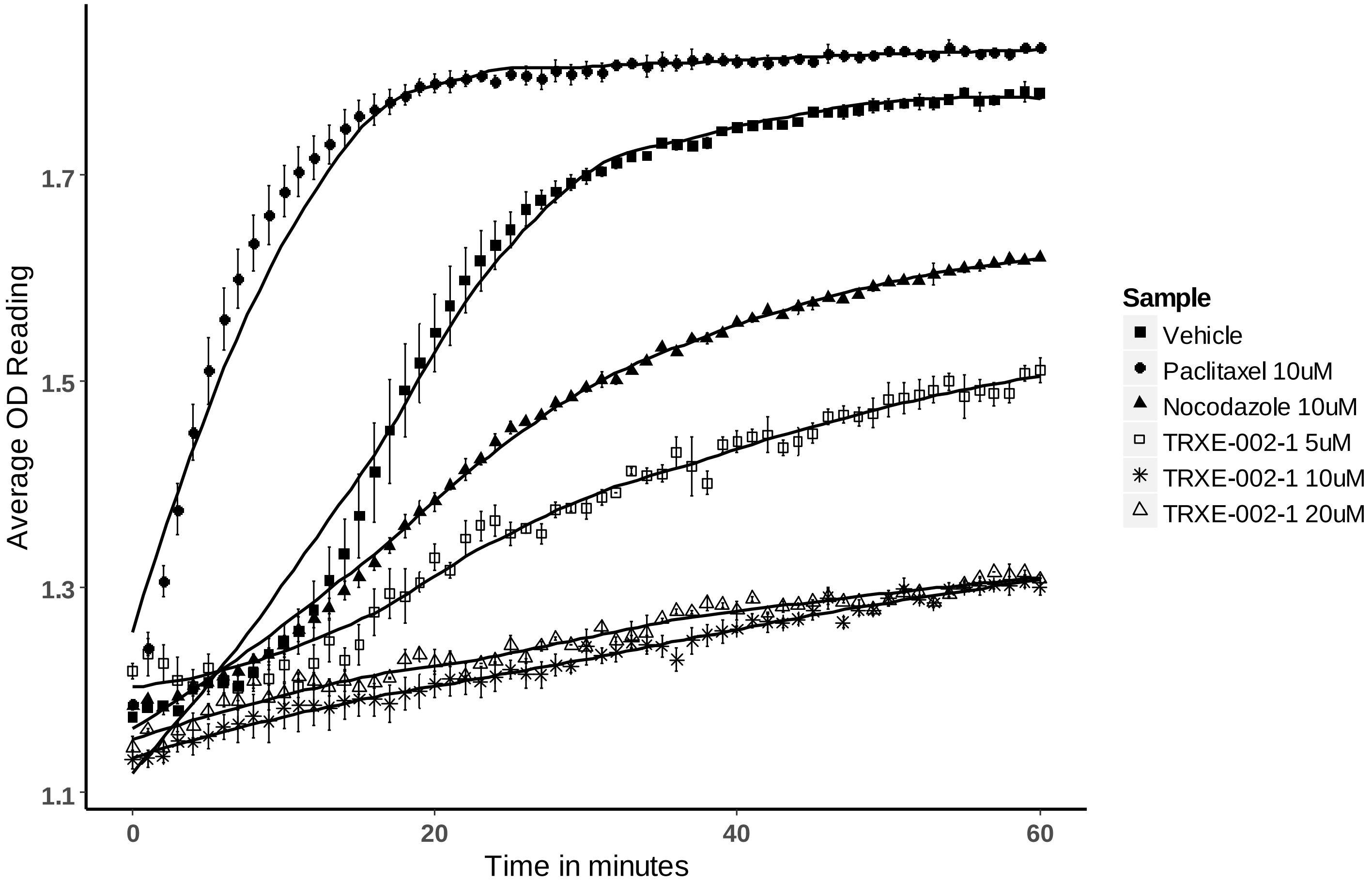

Supplementary Fig. S9B

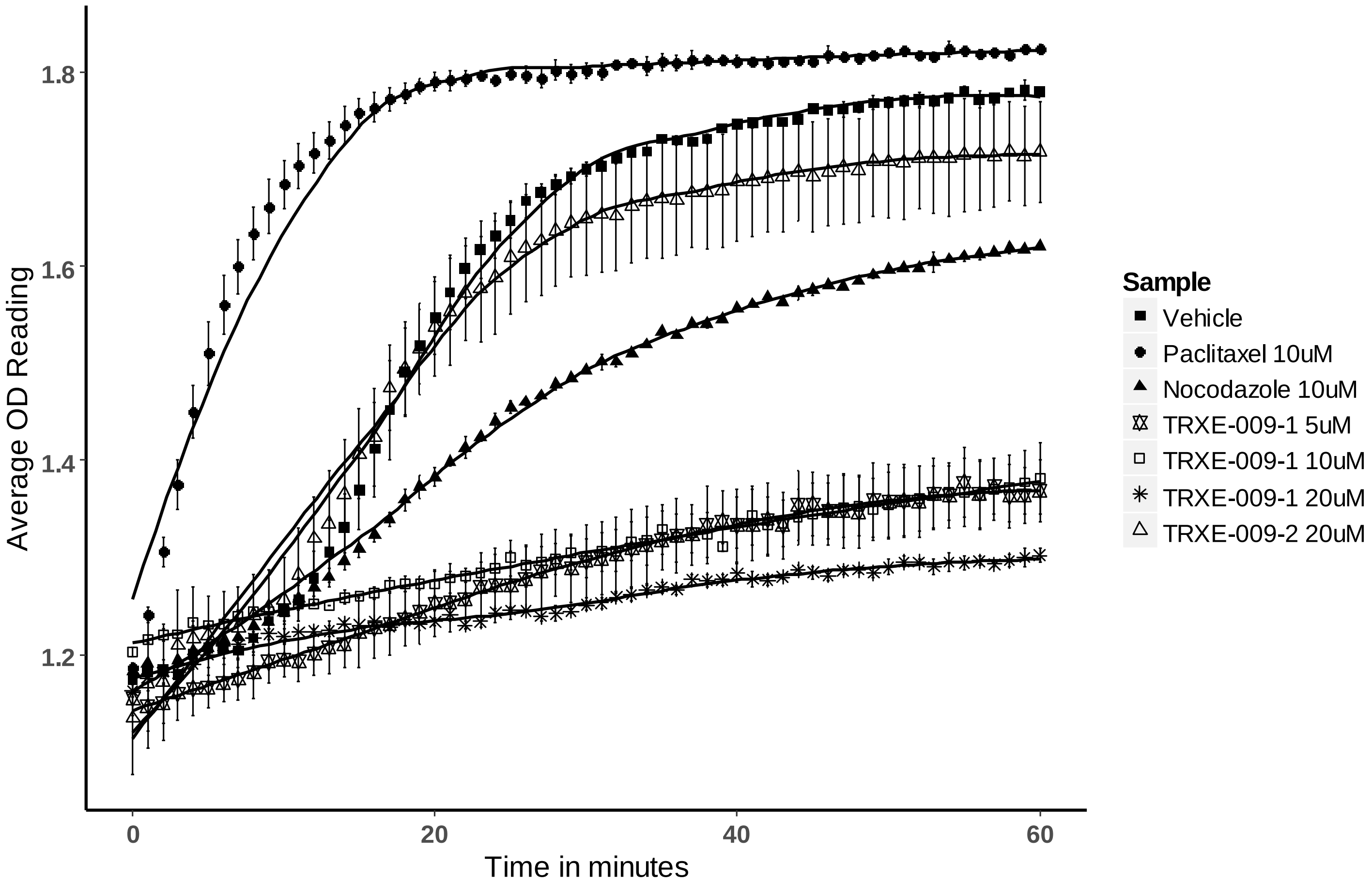

Supplementary Fig. S10A

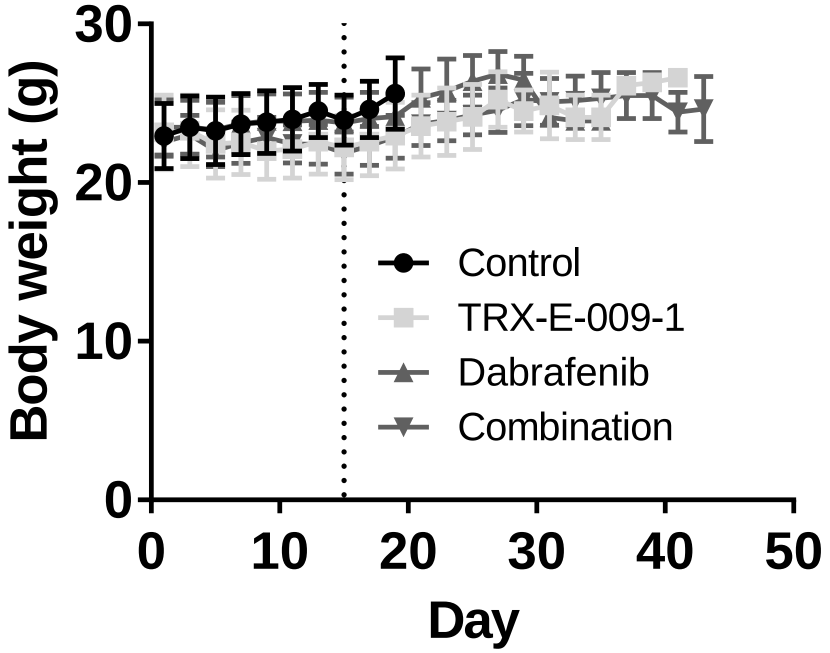

Supplementary Fig. S10B

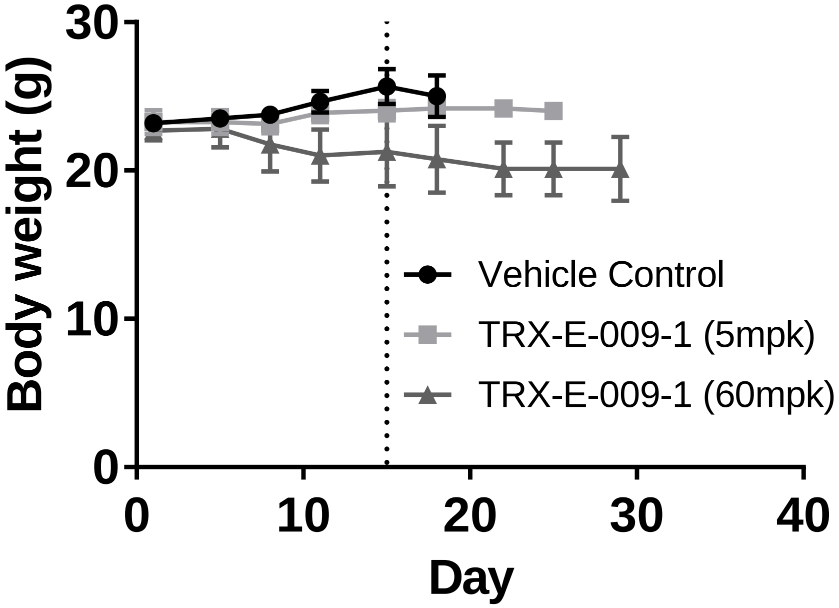

Supplement: Supplementary file 1 — Supplementary Data [file 41598_2018_22882_MOESM1_ESM.pdf]
